# The economic value of time of informal care and its determinants (The CUIDARSE Study)

**DOI:** 10.1371/journal.pone.0217016

**Published:** 2019-05-21

**Authors:** Juan Oliva-Moreno, Luz María Peña-Longobardo, Leticia García-Mochón, María del Río Lozano, Isabel Mosquera Metcalfe, María del Mar García-Calvente

**Affiliations:** 1 University of Castilla-La Mancha, Department of Economic Analysis and Seminar of Research on Economics and Health (SIES), Toledo, Spain; 2 Andalusian School of Public Health (EASP), Granada, Spain; 3 Biomedical Research Centre (ibs.GRANADA), Granada, Spain; 4 University of Basque Country, Department of Sociology and Research Group on social determinants of health and demographic change (OPIK), Gipuzkoa, Spain; University of Nebraska Medical Center, UNITED STATES

## Abstract

**Objective:**

The main aims of this paper are to analyse the monetary value of informal care time using different techniques and to identify significant variables associated with the number of caregiving hours.

**Data and methods:**

A multicentre study in two Spanish regions in adult caregivers was conducted. A total sample of 604 people was available. A multivariate analysis was performed to identify the variables associated with the number of hours of caregiving time. In the monetary valuation of informal care provided, three approaches were used: replacement cost method, opportunity cost and contingent valuation (willingness to pay and willingness to accept).

**Results:**

The main determinants of the amount of time of informal care provided were age, gender, the level of care receiver´s dependence and the professional care services received (at home and out of home). The value estimated for informal care time ranges from EUROS 80,247 (replacement cost method) to EUROS 14,325 (willingness to pay), with intermediate values of EUROS 27,140 and EUROS 29,343 (opportunity cost and willingness to accept, respectively). Several sensitivity analyses were performed over the base cases, confirming the previous results.

**Conclusions:**

Time of informal care represents a great social value, regardless of the applied technique. However, the results can differ strongly depending on the technique chosen. Therefore, the choice of technique of valuation is not neutral. Among the determinants of informal care time, the professional care received at home has a complementary character to informal care, while the formal care outside the home has a substitute character.

## Introduction

In the coming years, many European countries will have to face important demographic, technological and social challenges with profound changes in the current systems of care [1, 2]. This transformation has already started, although at different speeds among countries [3, 4]. In this way, Long-Term Care (LTC) systems are evolving into mixed models in which care is considered to be a shared responsibility, as opposed to models in which care is considered the responsibility of either the Welfare State or families [5, 6]. Non-professional care, also called informal care, is and will be a key element of LTC systems. Defining informal care is not straightforward [7, 8]. It encompasses a set of complex activities and tasks provided by non-professional people, normally by family, close relatives, friends or neighbours, whose objective is to help a person with limited autonomy to perform the basic or instrumental activities of her/his daily life. Informal caregivers usually have no limits on the time spent providing care. They do not usually receive any remuneration for their services although there can exist specific benefits, training and support programmes for carers in some countries [6, 9]. In sum, its most distinctive features are the non-professional character of caregiving and the affective relationship between the person being cared for and the caregiver.

One of the main challenges that LTC systems face is their financial sustainability. Some estimations point out that the public resources allocated to LTC systems might double, or even more, in the coming decades [10]. It is difficult to contemplate the sustainability of such systems without the intervention of informal care, since some authors maintain that up to 80% of long-term care could be provided by non-professional caregivers [11]. This does not mean that this resource is free. In this way, the opportunity cost of time spent by family carers may exceed the expenditure of formal care, especially in those countries such as Spain, where family is the backbone of LTC [12]. Such opportunity cost caused by family care is mainly associated with a reduction in labour force attachment for caregivers of working age as well as with a burden in terms of health and social problems suffered due to the care provided [13]. In fact, it has been supported that caregiving is undervalued socially and economically [14]. Therefore, not taking into consideration the opportunity cost of informal care might hide a relevant component that is part of the sustainability of LTC systems.

In fact, until recently, informal care had received little attention from policy makers [15] and has had low or even non-existent social recognition in many countries. However, the changes in the patterns of illness, with (i) a prolongation of life expectancy, (ii) significant number of elderly people with limitations, and (iii) the aforementioned demographic and social changes, have revealed, in an evident way, its current and future relevance [16]. For this reason, over the last 15 years, the recognition and the formalisation of the role played by informal carers (thought training opportunities, social security, legal rights and/or cash payments) has been one of the main trends within LTC system in Europe [17], helping to ensure the sustainability of caregiving overall. Therefore, recognising the informal carer`s needs as well as pointing out its monetary value, is one of the current priority for LTC policies.

The inclusion of informal care in the field of cost of illness studies is increasingly frequent in those diseases that cause a high degree of dependence such as Alzheimer disease, stroke, rare disease and mental illness, among others, revealing its economic relevance from the societal perspective [18, 19]. On the other hand, in the field of the economic evaluation of health care interventions, the inclusion of informal care is strongly conditioned by the rules that each country adopted or recommended [20]. However, in a context of population aging and with an increasingly important presence of chronic diseases in the demand for health services and in the global burden of diseases, their inclusion is compulsory for the aforementioned diseases if the perspective employed is the societal one.

At the macro level, some studies have tried to estimate the aggregated value of informal care at the national level [21–25]. The results of these studies conducted in the USA, Canada, France and Spain confirmed that family caregiving has a highly significant economic impact at an aggregated level. These results are robust in spite of the differences in the methodological issues and the asymmetric development of formal long-term care systems among countries. However, there is not a unique approach agreed upon the assessment of informal care. In fact, very few papers have employed different techniques to estimate the value of the care provided by informal caregivers. Therefore, the goal of this paper is twofold. First, we will identify the variables associated with the amount of informal care time, measured in terms of time of care provided by a sample of informal caregivers. Secondly, we will value this caregiving time using the three most frequently used techniques in the field of health economics: opportunity cost, replacement cost and contingent valuation.

## Data and methods

### Data and variables

Data are from a cross-sectional epidemiological study carried out in 2013 in the adult caregiver population in two geographical areas of Spain: the province of Granada (Andalusia) and the province of Gipuzkoa (Basque Country). The data correspond to the first temporal wave of a multicentre longitudinal study (CUIDAR-SE Study). All participants provided informed consent and it was written.

The study population was made up of people aged 18 or above living in a family who informally (not professionally) gave care to a person in a situation of dependence, regardless of whether they lived together or not. These persons had to be registered as caregivers in the registers of the Primary Health Care District of Granada or of the Social Services of the Provincial Council of Gipuzkoa. Thus, in Granada, participants had been included in a registry of caregivers when they contacted primary health care services. In Gipuzkoa, participants had been included in a registry of social services because of having requested some service from those offered to caregivers.

The data collection process began in September 2013 and ended in December of the same year. Personal interviews were conducted with a structured questionnaire designed ad hoc (see S1 File). The questionnaire was designed based on the available literature on the object of study, a specific search for services and actions aimed at caregivers in the field studied, and the scales and instruments validated in our context [26]. The questionnaire included variables on the characteristics of the caregivers, such as age, gender, educational level (“1” stands for no studies, “2” for primary and secondary education, and “3” for tertiary education), region, health related quality of life (HRQoL) (measured through the EQ5D-5L proxy questionnaire which defines quality of life through 5 dimensions: mobility; self-care; everyday activities; pain/discomfort; and anxiety/depression. The values or utilities are indicated on a scale where “0” corresponds to death and “1” corresponds to perfect health, with negative values being possible), level of dependency (categorized into moderate: when the person needs help to perform several basic activities of daily living, at least once a day or has intermittent or limited support needs for their personal autonomy; severe: when the person needs help to perform several basic activities of daily life two or three times a day, but does not want the permanent support of a caregiver or has extensive support needs for their personal autonomy; major: when the person needs help to perform several basic activities of daily life several times a day and, because of their total loss of physical, mental, intellectual or sensorial autonomy, needs the indispensable and continuous support of another person or has generalized support needs for their personal autonomy), whether the person receives social support, whether the person receives economic benefits, whether he/she receives services at home (home help care, telecare and nurses services) or outside (day centres, residences and helping and training courses), and how caregivers face the care provided (whose value is “1” if the caregiver refuses to believe that caregiving happened, and “0” otherwise). Regarding information on the care provided, the information refers to the years of care and the number of daily caregiving hours provided. All this information was presented in a descriptive analysis in which t-tests to compare caregiver characteristics by sex were also performed.

The questionnaire, previously piloted, was applied through a personal interview at the home of the caregivers. All the selected persons received a letter from those in charge of the health district of Granada or the Provincial Council of Gipuzkoa, inviting them to participate in the study and explaining the objectives of the study and all the ethical and confidentiality aspects associated with their participation, indicating that (1) all participants consented, (2) consent was informed.

### Analysis of informal care time

To analyse the main factors associated with the time of informal care (that is, the number of informal caregiving hours), a multivariate regression model (ordered logistic model) was used. According to the distribution of the caregiving time provided, the dependent variable was classified in three categories; “high” if the hours of weekly care provided were less than 70 (10 hours per day-7 days per week); “extremely high” if the weekly hours of care ranged between 70 and 130; and “constant” if the informal care provided was higher than 130 hours per week.

The specification of the model is as follows:

$$prob_{i}(timej)\;=\;Ʌ(\alpha_{1}-\;\beta {´}_{1}X_{i}-\;\varepsilon_{i})$$

$$prob_{i}(timej)\;=\;Ʌ(\alpha_{j}-\;\beta {´}_{j}X_{i})-\;Ʌ(\alpha_{j-1}-\beta {´}_{j-1}X_{i})-\;\varepsilon_{i},$$

$$j\;=\;2,\;\ldots ,\;j-1prob_{i}(timej)\;=\;1-\;\sum_{j\;=\;1}^{j-1}prob_{i}(timej)$$

Where prob_i_ (time_j_) is the probability that subject i (i = 1, …, I) belongs to the category of the time of informal care provided that takes values j = 1, 2 and 3; Ʌ denotes the logistic distribution function; X_i_ represents the vector of explanatory variables which are age, gender, educational level, region, health related quality of life (HRQoL), level of dependency, whether the person receives social support, whether the person receives economic benefits, whether he/she receives services at home or outside, and how caregivers face the care provided; β is the vector of coefficients parameters assigned to each explanatory variable included in the vector X; and ε_i_ is the standard error.

More specifically, the extended specification of the model is the following:

$$\begin{array}{l} Leveloftimei\;=\beta_{0}+\beta_{1}{\text{middlehighage}}_{\text{i}}+\beta_{2}{\text{highage}}_{\text{i}}+\beta_{3}{\text{female}}_{\text{i}}+\beta_{4}{\text{primarystudies}}_{\text{i}}+\beta_{5}secondarystudies_{i}+\beta_{6}Granada_{i}+\beta_{7}highHRQoL_{i}\\ +\;\beta_{8}moderatedependence_{i}+\;\beta_{9}severedependence_{i}+\;\beta_{10}socialsupport_{i}+\;\beta_{11}financialbenefits_{i}+\;\beta_{12}socialservicesinhome_{i}\;\\ +\;\beta_{13}socialservicesouthome_{i}+\;\beta_{14}otherservices_{i}+\;\beta_{15}negativecoping_{i}+u_{i} \end{array}$$

### Economic assessment of time of informal care

Valuation of care time can be calculated using different methods [7, 27–30]. As informal care is not offered in the market and there is no market price for it, it is necessary to allocate a shadow price for the valuation of the time of care. Particularly, in order to assess the value of caregiving hours, different techniques were used. Firstly, in the replacement cost method (also called proxy good method) the time spent on informal care is assessed through the (labour) market prices of a close market substitute. We used the price of the home help services provided by the Instituto de Mayores y Servicios Sociales (IMSERSO) for the regions of Andalusia and the Basque Country in 2013 as a reference year [31]. The hourly shadow prices used were EUR 13.0 and EUR 19.06 for Granada (Andalusia) and Gipuzkoa (Basque Country), respectively. We considered an alternative and conservative scenario (Scenario 1) as part of the sensitivity analysis, applying the lowest shadow price (EUR 13.0) in both regions.

The second technique used for evaluating the hours of care was the opportunity cost method. This method evaluates the time invested in providing informal care, taking into account the best alternative that caregivers have to give up due to the informal caregiving services provided. It takes into account three shadow prices: paid working time, unpaid working time and leisure time. However, due to the lack of works on the valuation of leisure time in Spain, we applied the same shadow price to the leisure time as to unpaid working time [31]. For unpaid working time and leisure time, the minimum wage of a domestic employee was used for the year 2013 in Spain (EUR 5.05 per hour). For the assessment of paid working time, hours were valued with the wage provided by the Salary Structure Survey of the National Institute of Statistics for Andalusia and the Basque Country in 2013, which was EUR 14.12 and EUR18.41 per hour, respectively. Different assumptions were applied for valuating paid working hours. Firstly, for those who declared that they had left work permanently or temporarily, we considered that the time of care was valued as paid working time if such time was less than 37.5 hours per week. Conversely, if the caregiving time was higher than 37.5 h/week, then 37.5 hours were considered as paid work and the rest was considered as unpaid work and leisure. Thereby, for those individuals who indicated that they were still working but had to reduce working time due to the care provided, it was assumed that they had reduced 2 hours of work per day. As a sensitivity analysis, we estimated the opportunity cost assuming 3 (Scenario 1) and 1.5 (Scenario 2) daily working hours reduction.

The last technique used was the contingent valuation method (CVM), which simulates a hypothetical market through surveys of actual or potential caregivers. The objective of such technique is to present a reliable scenario for the surveyed people, in such a way that they reveal their willingness to pay (WTP) or their willingness to accept (WTA) for the implementation (or withdrawal) of a program that influences the number of hours of care provided. The identification of the WTP and the WTA was done using questionnaires specifically designed for this purpose.

The WTA for providing an additional daily hour of attention to the person already cared for was raised under a hypothetical scenario in which the State could offer an economic compensation in exchange for that added time. To be able to answer, a payment card format was used, in which each person was randomly shown the following amounts of money in euros per day: EUR 0, EUR 1, EUR 2, EUR 3, EUR 4, EUR 5, EUR 6, EUR 8, EUR 10, EUR 12 and EUR 15. The amount of money equivalent to euros per month was also presented in the same format, so that the respondent could express their answers in either of the two forms, euros per day or euros per month. For each payment card shown, they were asked to form three groups, depending on whether they considered the amount shown in them to be: (i) enough compensation; (ii) insufficient compensation; (iii) do not know if it would be enough compensation or not. Likewise, those people who answered that a compensation of EUR 15 / day (EUR 450 per month) was an insufficient amount were asked to indicate the minimum daily (or monthly) amount they considered enough to be compensated or compensate for caring for that person for one more hour a day. On the other hand, those people who indicated that a compensation of EUR 0 would be insufficient, they were additionally asked about the reasons underlying their response.

The estimation of the WTP for informal care was carried out in a manner very similar to the WTA. In this case, the question was focused on determining the maximum WTP in the hypothetical scenario in which the State would provide the assistance of a professional caregiver for the care of the person who was already care for, but in return the carer would have to assume a copayment.

Additionally, the existence of “protest zeros” and “economic zeros” due to severe budget constraints was considered. In these cases, when the response is zero, it does not mean that the real value that the person gives to one hour of care is zero. Therefore, as part of the sensitivity analysis, the values of these zero protests were replaced by the average WTA and the average WTP of the sample taking into consideration the age, the region and the level of education. More precisely, in the case of obtaining a response with a value of EUR 0, the reasons derived from this response were examined, distinguishing between “zero protest" and real zero values. In this way, the zero values considered as “true” values were those classified as such according to the following reasons: "Caring for that person one hour more per day would not mean a difference so big as to need to be compensated for" (WTA), or "taking care of that person one more hour a day would not be an important difference and, therefore, it would be indifferent to me whether a professional caregiver came or not"(WTP). On the contrary, when the response was justified for one of the following reasons "It is a matter of principle, I would feel bad accepting money in exchange for the caregiving of that person." (WTA), "it is a matter of principle, I would not pay a single euro for a public service" or "I do not trust professional caregivers” (WTP) the valuation was considered a "zero protest”, because these answers denotes rejection or protest to the proposed scenario due to ethical objections.

It should be noted that, in the assessment of care time, as a conservative criterion, we have censored the time of care to a maximum of 16 h per day when the time of care reported exceeded this figure, considering that caregivers can perform several tasks simultaneously or that they may be available at night, but also devote time to sleep (8 hours), which is consistent with the approach adopted in other studies [25, 32–36]. Base year was 2013 and the assessment of time was considered in annual terms to facilitate comparisons with the cost of illness studies in the literature.

## Results

Table 1 shows the main sociodemographic characteristics of caregivers considered in the analysis. More than 56% of the caregivers were female and the average age was 59.8 years. Likewise, around 40% of carers had no studies completed, 25.9% had primary education and 34% secondary or tertiary education and almost 88% of them cared for individuals with severe or major dependence. Their HRQoL was 0.827 over 1 and almost 5% of them coped negatively with the care provided. More than 85% of individuals who received care had social services at home, 17.38% received services out of home and more than 79% had monetary benefits. This table also provides caregivers’ information separately by gender. Thereby, figures show that male caregivers were older than females (62 vs 57 years old). Moreover, it is also identified statistical differences in the percentage of caregivers who cared for individuals with monetary benefits, being those individuals cared by females the ones who received a higher proportion of such benefits. Finally, male carers provided more weekly caregiving hours (122 hours per week vs 113 h per week, respectively).

**Table 1:** Sociodemographic characteristics of caregivers.

	Total (n = 610)	Male(n = 265)	Female (n = 345)	Comparison of means p-value
	Average (SD) or %	Average (SD) or %	Average (SD) or %	
Gender (female)	56.56	-	-	-
Age (mean, SD)	59.82 (14.47)	62.28 (16.28)	57.94 (12.62)	0.001
Education				
No studies completed (yes)	40.07	45.08	36.23	0.184
Primary Education (yes)	25.94	21.21	29.57	
Secondary/Tertiary Education (yes)	33.99	33.71	34.20	
Level of Dependence				
Moderate (yes)	12.24	13.18	11.46	0.075
Severe (yes)	57.34	61.24	54.14	
Major (yes)	30.42	25.58	34.39	
HRQoL (mean, SD)	0.827 (0.194)	0.836 (0.203)	0.821 (0.187)	0.323
Granada (yes)	51.31	50.19	52.17	0.627
Formal Services				
Services at home (yes)	85.74	84.15	86.96	0.326
Services out of home (yes)	17.38	13.96	20.00	0.051
Monetary benefits (yes)	79.51	73.96	83.77	0.002
Other services (yes)	66.56	66.04	66.96	0.811
Coping with caregiving (negative)	4.92	4.91	4.93	0.990
Weekly time of informal care (mean, SD)	117.10 (42.13)	122.36 (39.55)	113.08 (43.64)	0.007
Weekly time of informal care (censorship) (mean, SD)	96.56 (25.38)	99.86 (22.74)	94.04 (26.98)	0.005

Source: authors ‘analysis from the CUIDAR-SE study

Table 2 shows the results obtained from the ordered logit model which displays the main determinants of caregiving hours. Thus, gender, age, region, level of dependence as well as the social services received are the main factors associated with the amount of caregiving time received. Thereby, women had 9.6 percentage point less probability of providing a high amount care (more than 130 hours of care per week). Likewise, those carers who were older than 65 years had 17.2 percentage points higher probability of providing a high amount of care compared to those caregivers younger than 50 years old. Besides, caregivers living in Granada had 26 percentage points lower probability of providing more than 130 hours of care per week compared to those living in Gipuzkoa. Likewise, those who cared for individuals with major dependence had 17.4 percentage points higher probability of providing more than 130 hours of care per week, compared to those who cared for individuals with moderate dependence. Finally, those caregivers who cared for those receiving social services at home had 13.6 percentage points more probability of providing a high amount of informal caregiving while those who care for individuals with social services out of home had 18.2 percentage points less probability of providing informal care. Such results indicate that services received at home are complementary to family care, while services received out of home are substitutes to informal care.

**Table 2:** Determinants of informal care time. Results from the ordered logistic model.

	Average Marginal Effects^1^ (Constant)		Average Marginal Effects^1^ (Extremely-High)		Average Marginal Effects^1^ (High)	
	dy/dx	P value	dy/dx	P value	dy/dx	P value
Age (50–64 years old)	0.063 (0.050)	0.209	-0.027 (0.022)	0.233	-0.036 (0.028)	0.197
Age (≥65 years old))	**0.172** **(0.057)**	**0.003**	**-0.077** **(0.029)**	**0.008**	**-0.094** **(0.030)**	**0.002**
Female	**-0.096** **(0.039)**	**0.016**	**0.040** **(0.018)**	**0.027**	**0.055** **(0.022)**	**0.014**
Secondary education	0.023 (0.053)	0.657	-0.010 (0.023)	0.667	-0.013 (0.030)	0.649
Tertiary education	-0.045 (0.050)	0.371	0.017 (.019)	0.352	0.027 (0.031)	0.387
Granada	**-0.260** **(0.044)**	**0.001**	**0.106** **(0.023)**	**0.001**	**0.153** **(0.027)**	**0.001**
High HRQoL^2^	-0.035 (0.043)	0.411	0.015 (0.018)	0.425	0.020 (0.024)	0.403
Severe Dependence	0.109 (0.078)	0.162	-0.047 (0.036)	0.190	-0.061 (0.042)	0.148
Major Dependence	**0.174** **(0.073)**	**0.018**	**-0.067** **(0.028)**	**0.017**	**-0.107** **(0.047)**	**0.025**
High social support	0.003 (0.048)	0.943	-0.001 (0.019)	0.942	-0.002 (0.028)	0.943
Monetary Benefits	0.070 (0.048)	0.140	-0.025 (0.014)	0.089	-0.045 (0.034)	0.179
Social services at home	**0.136** **(0.048)**	**0.005**	**-0.035** **(0.010)**	**0.001**	**-0.100** **(0.034)**	**0.025**
Social services out of home	**-0.182** **(0.040)**	**0.001**	**0.039** **(0.012)**	**0.002**	**0.142** **(0.041)**	**0.001**
Other services	0.070 (0.044)	0.111	-0.026 (0.015)	0.094	-0.044 (0.029)	0.131
Face to care (negative)	0.163 (0.094)	0.084	-0.089 (0.061)	0.150	**-0.074** **(0.033)**	**0.027**
N	581					
LR chi2	94.76					
Pseudo R2	0.0778					

^1^ Predicted probabilities based on the model.

^2^ It takes “1” for score being equal or higher than 0.85 points over 1. “0” otherwise. Source: authors ‘analysis from the CUIDAR-SE study

Regarding the assessment of caregiving time (Table 3), the number of weekly hours of care ranged between 96.6 and 117.1 hours (between 5,035 and 6,106 annually hours), depending on whether the hours were censored or not (Table 1). The highest valuation resulted from using the replacement cost method, with an annual valuation (without censure) of EUR 80,247 per caregiver (EUR 98,136). This means that the unit value of care time stands at EUR 15.9 (EUR 16.1) per hour of care. The economic value was lower when applying the opportunity cost method, with a total annual valuation (without censorship) of EUR 27,140 per caregiver (EUR 32,512). The reason for this lower valuation is due to the unit value of care time applied in this method, which stands at EUR 5.4 euros (EUR 5.3) per hour of care. This is because a great part of the time of care is unpaid work-leisure. Given that this use of time has been valued very conservatively (EUR 5.05 per hour), this explains the differences between the valuation achieved by this method and the replacement cost one. Third, the valuation achieved through contingent valuation techniques led us to annual figures ranging from EUR 14,325 per year and per caregiver (WTP) to EUR 29,343 (WTA) (EUR 17,577 and EUR 33,744 without censorship). Thus, the unit values of informal care range between EUR 2.84 (WTP) and EUR 5.83 (WTA) (EUR 2.88 and EUR 5.53, respectively, without censorship). The consideration of protest zeros and economic zeros modifies only slightly the assessments of total and censored care time, given the limited number of these cases.

**Table 3:** Economic valuation of informal care (annual) per caregiver.

Main estimation (censorship applied to 16 daily hours of informal care as maximum)					
	**N**	**Mean**	**SD**	**Min**	**Max**
Economic valuation. Replacement method (with censorship). Base case.	604	80,247.33	26,430.9	12,879.3	111,310.4
Economic valuation. Opportunity cost method (with censorship). Base case.	604	27,140.07	8,228.37	3,686.5	53,540
Economic valuation. Contingent Valuation (WTP) (with censorship). Base case.	539	14,325.11	25,652.0	0.0	175,200.0
Economic valuation. Contingent Valuation (WTA) (with censorship). Base case.	523	29,343.32	39,068.8	0.0	292,000.0
Sensitivity analysis (I)					
	**N**	**Mean**	**SD**	**Min**	**Max**
Economic valuation. Replacement method (with censorship). Scenario 1	604	65,460.35	17,204.21	9,490	75,920
Economic valuation. Opportunity cost method (with censorship). Scenario 1.	604	27,219.51	8,286.13	3,686.5	53,540
Economic valuation. Opportunity cost method (with censorship). Scenario 2.	604	27,046.33	8,214.48	3,686.5	53,540
Economic valuation. Contingent Valuation (WTP) (with censorship) (including estimation of real values of zeros protest and economic zeros)	549	15,452.55	25,535.03	0.0	175,200.0
Economic valuation. Contingent Valuation (WTA) (with censorship) (including estimation of real values of zeros protest)	565	30,730.86	38,269.24	0.0	292,000.0
Sensitivity analysis (II). Valuation of informal care without censorship					
	**N**	**Mean**	**SD**	**Min**	**Max**
Economic valuation. Replacement method (without censorship). Base case.	604	98,135.78	42,949.6	12,879.3	166,965.6
Economic valuation. Proxy good method (without censorship). Scenario 1.	604	79,382.23	17,204.21	9,490	113,880
Economic valuation. Opportunity cost method (without censorship). Base case.	604	32,512.18	12,020.15	3,686.5	68,286
Economic valuation. Opportunity cost method (without censorship). Scenario 1.	604	32,627.66	12,040.33	3,686.5	68,286
Economic valuation. Opportunity cost method (without censorship). Scenario 2.	604	32,454.45	12,020.39	3,686.5	68,286
Economic valuation. Contingent Valuation (WTP) (without censorship)	539	17,577.22	32,918.0	0.0	190,842.9
Economic valuation. Contingent Valuation (WTA) (without censorship).	523	33,744.26	44,363.2	0.0	310,250.0
Economic valuation. Contingent Valuation (WTP) (including estimation of real values of zeros protest and economic zeros) (without censorship).	549	18,957.05	32,789.4	0.0	190,842.9
Economic valuation. Contingent Valuation (WTA) (including estimation of real values of zeros protest) (without censorship).	565	35,262.27	43,507.8	0.0	310,250.0

Scenario 1: assuming 3 daily working hours reduce. Scenario 2: assuming 1.5 daily working hours reduce Source: own elaboration from The CUIDAR-SE study

## Discussion

This study analyses the monetary value of informal caregiving in two different regions of Spain. This assessment has been carried out applying different techniques, which provide very different figures depending on the approach considered.

Although the selection of carers was not carried out looking for representation at the national level, our results show the high number of informal caregiving hours for family members and friends of the caregivers interviewed. In this sense, it should be stressed that there is a high heterogeneity between OECD countries in the provision of such service. For instance, countries such as Spain, Portugal or Poland have the highest rate in the amount of care provided by informal caregivers, where more than 50% of informal caregivers provided more than 20 hours weekly, while in countries such as Denmark or Norway only 9% of carers provided such an amount of caregiving hours [10]. This is a very relevant issue to be considered when taking into account the composition of the LTC system in Spain, in which family plays a dominant role as it is the safest care provider to cover the needs of people in situations of dependency, while the public-sector support is secondary [37]. In this sense, in 2006, the Law of Promotion of Personal Autonomy and Assistance for Persons in a Situation of Dependency (SAAD) was established in Spain in the framework of a LTC system to provide care to people with difficulties in carrying out daily life activities. However, instead of offering services in-kind, the monetary benefits from providing care to their relatives have become a common practice because they are not only more affordable than investing on or contracting out service provisions, but also because cash benefits are preferred by families (they represent an income entry for the household and they do not have to bear the co-payment for in-kind services) [37]. Then, due to the high number of hours of caregiving, the composition of the LTC system as well as cultural aspects, the care provided by family members plays a fundamental role within the LTC system in Spain, and, therefore, it needs to be considered when designing policies focused on personal care of people with limitations in their activities of daily living. However, this is not limited to the Spanish sphere, but is extended to all of Europe, since the traditional distinction between professional and non-professional (informal) care is being transformed in the context of the evolution of long-term care systems [38].

In this sense, the literature has studied the relationship between formal and informal care from several alternative ways. Firstly, the compensatory model holds that caregivers may lean on formal care when no other alternatives are available. This model establishes that the family is the first group in terms of importance in the support of people who need it and that the patterns of assistance follow a hierarchical order determined by the preferences of the individuals. Each of the groups -first the family, followed by friends and neighbours, and finally the formal services-, provides assistance when the previous preferred sources of support are not available (substitution model) or are insufficient (complementarity model). Finally, the task specific model considers that what determinates the type of care provision is the nature of the task (informal care is usually used for tasks in which an affective relationship is present as well as in tasks of supervision care) [39, 40]. Our estimates actually indicate that services received at home are complementary to informal care while services received out of home are substitutes to informal care. This conclusion is in line with the previous literature in which different assumptions about the relationship between formal and informal care are considered [41].

Moreover, taking into consideration all the aforementioned assumptions, the literature reveals that the relationship between these two types of care would depend on the health status of the person who is cared for (such as the diseases suffered from), tasks needed, the relationship between the carer and the dependent person, household income and sociocultural factors [42–47]. Our statistical analysis reveals that the main variables associated with the amount of caregiving time provided were gender, age, region, level of dependence as well as the reception of social services. On the other hand, contrary to the literature that indicates more intensive care in women [48–50], our study indicates that women have a lower probability of caring intensively than men. Possibly this could be due to the fact that the profile of male caregivers of the sample are mostly older men who take care of their wives at home, while women have a more heterogeneous profile [13].

Another relevant result from our analysis confirms that the election of the valuation method applied has a considerable influence in the result as the average cost ranged from EUR 80,247 per year (when considering replacement method) to EUR 14,325 (WTP), going through EUR 27,140 (opportunity cost method) and EUR 29,343 (WTA). This high disparity in the value of informal caregiving time implies that the choice of the valuation method is not neutral. A recent systematic review on cost of illness studies that included informal care costs revealed that the opportunity cost method is present in almost 69% of the studies and the replacement method in 27% of them [18]. So, if the election of the approach has a strong influence on the estimated values of the cost of a disease, it could also influence the results of the economic evaluations of health interventions in diseases that require intensive informal care.

In any case, and even using the most conservative estimates, the results indicate the great importance of informal caregivers in Spain as a care network for people with limited autonomy. This can be proved by comparing the results of our estimates with the public prices (tariffs) of professional (formal) care published by Spanish public institutions [31]. Thus, the average annual price of a place in a day centre in Spain was estimated at EUR 8,750 (EUR 15,500 in the Basque Country and EUR 7,300 in Andalusia), while the annual price of a residence place amounted to EUR 18,000 (EUR 23,000 in the Basque Country and EUR 17,000 in Andalusia). This means that the estimated annual opportunity cost of informal care estimated in our work exceeds the cost of a residential place. On the other hand, the public price of a residence is much lower than the estimated value of informal care using the replacement cost, although we should consider that the cost of replacement is established based on a home care service while the residential place would mean changing the environment of the person cared for.

Considering the great importance of informal care in Spain, as well as the predictions stating that future demand of informal care will exceed the supply [51, 52], a prompt replacement of informal care for professional services would collapse the long-term care system and make it financially unsustainable. Therefore, it is essential to consider the current role of informal care not only in Spain but also in most European countries in order to evolve towards a LTC system that provides much more support to informal caregivers and where the transition between informal care and formal care is carried out in a smooth and programmed manner.

Some limitations should be highlighted. Firstly, the survey used is a cross-sectional and not longitudinal data. A consequence of such limitation is the impossibility of establishing a causal association between the caregiving time and the elements analysed. Secondly, the sample involves carers living in two different regions of Spain. Therefore, it is not a representative at the national level. Consequently, caution is needed when extrapolating such results to the entire population or population caring for individuals with specific diseases. Third, when estimating the value of informal care using the opportunity cost approach, the shadow price applied to leisure time has been the same as that applied to unpaid production time. The absence of Spanish studies on the value of leisure time has advised acting in this way. Finally, with respect to the methods of assessment of informal care, each of them has advantages and limitations [7], so there is no single commonly accepted method of assessment. This is not a limitation proper of this work and suggests that, in the estimation of the value of informal care, it is useful to consider the application of several methods to enrich the analysis. Finally, it should be mentioned that all the individuals from Gipuzkoa included in the sample received some social/formal attention services, and this might influence the results. In fact, a possible limitation of the study is that caregivers who have not had any contact with health or social services have not been included. It is likely that this missing profile corresponds to people with less involvement in care. In any case, we consider that the profile that we collected in this study is that of caregivers with a high dedication to care. Therefore, the results could be extrapolated to this caregiver profile.

Several future lines of research arise from the results of the present study. Firstly, when the amount and the value of time caregiving are analysed, it would be interesting to find out whether family care causes an effect on caregivers, in terms of burden and other impacts (in three different dimensions: physical and mental health, professional and social life) because of the tasks carried out. Moreover, due to the differences found in the value of WTA and WTP, it would be convenient to analyse in detail the main factors that explain the valuations reaches with each of these contingent valuation technique.

## Supporting information

S1 File This is the S1 file questionnaire. (PDF)
