# Supplementary material for: The economic value of time of informal care and its determinants (The CUIDARSE Study)
Source: PLoS One. 2019 May 21;14(5):e0217016. doi: 10.1371/journal.pone.0217016 (PMC6529156; doi:10.1371/journal.pone.0217016)
Supplement: S1 File — (PDF) [file pone.0217016.s001.pdf]

Escuela Andaluza de Salud Pública
CONSEJERÍA DE SALUD Y FAMILIAS

EUSKO JAURLARITZA
GOBIERNO VASCO
OSASUN SAILA
DEPARTAMENTO DE SALUD

QUESTIONNAIRE OF CUIDAR-SE PROJECT:

LONGITUDINAL STUDY OF WOMEN AND MEN CAREGIVER'S HEALTH AND QUALITY OF LIFE IN ANDALUSIA AND BASQUE COUNTRY (SPAIN)

This questionnaire has been designed ad hoc by the research team of the CUIDAR-SE Project. The project has a multicentric nature, is developed in both Granada and Gipuzkoa (Spain). It is a longitudinal project and, therefore, the questionnaire has been designed to collect the information every 12 months from the same caregivers. This questionnaire corresponds to the initial wave of 2013.

Cite as:

María del Mar García Calvente, María del Río Lozano, Isabel Larrañaga Padilla. Cuestionario del Proyecto CUIDAR-SE: Estudio de seguimiento de la salud y la calidad de vida de mujeres y hombres cuidadores en Andalucía y País Vasco. Granada: Escuela Andaluza de Salud Pública; 2019.

Address for correspondence: maria.rio.easp@juntadeandalucia.es

María del Mar García Calvente, María del Río Lozano, Isabel Larrañaga Padilla. Cuestionario del Proyecto CUIDAR-SE: Estudio de seguimiento de la salud y la calidad de vida de mujeres y hombres cuidadores en Andalucía y País Vasco. Granada: Escuela Andaluza de Salud Pública; 2019.
Financiado por el Ministerio de Economía y Competitividad, Instituto de Salud Carlos III y Fondos FEDER (Expte. Nº PI12/00498).

MINISTERIO DE ECONOMÍA Y COMPETITIVIDAD
Instituto de Salud Carlos III
Unión Europea
Fondo Europeo de Desarrollo Regional
"Una manera de hacer Europa"
Andalucía se mueve con Europa

Escuela Andaluza de Salud Pública
CONSEJERÍA DE SALUD Y FAMILIAS

EUSKO JAURLARITZA
GOBIERNO VASCO
OSASUN SAILA
DEPARTAMENTO DE SALUD

CUESTIONARIO DEL PROYECTO CUIDAR-SE: ESTUDIO DE SEGUIMIENTO DE LA SALUD Y LA CALIDAD DE VIDA DE MUJERES Y HOMBRES CUIDADORES EN ANDALUCÍA Y PAÍS VASCO (ESPAÑA)

Este cuestionario ha sido diseñado ad hoc por el equipo de investigación del Proyecto CUIDAR-SE. El proyecto tiene un carácter multicéntrico, se desarrolla en Granada y Gipuzkoa. Es un proyecto se seguimiento y, por ello, se ha diseñado el cuestionario para recoger la información cada 12 meses de las mismas personas cuidadoras. El presente cuestionario corresponde a la oleada inicial de 2013.

Citar como:
María del Mar García Calvente, María del Río Lozano, Isabel Larrañaga Padilla. Cuestionario del Proyecto CUIDAR-SE: Estudio de seguimiento de la salud y la calidad de vida de mujeres y hombres cuidadores en Andalucía y País Vasco. Granada: Escuela Andaluza de Salud Pública; 2019.

Dirección de correspondencia: maria.rio.easp@juntadeandalucia.es

María del Mar García Calvente, María del Río Lozano, Isabel Larrañaga Padilla. Cuestionario del Proyecto CUIDAR-SE: Estudio de seguimiento de la salud y la calidad de vida de mujeres y hombres cuidadores en Andalucía y País Vasco. Granada: Escuela Andaluza de Salud Pública; 2019.
Financiado por el Ministerio de Economía y Competitividad, Instituto de Salud Carlos III y Fondos FEDER (Expte. Nº PI12/00498).

MINISTERIO DE ECONOMÍA Y COMPETITIVIDAD
Instituto de Salud Carlos III
Unión Europea
Fondo Europeo de Desarrollo Regional
"Una manera de hacer Europa"
Andalucía se mueve con Europa

ESTRUCTURA DEL CUESTIONARIO:

BLOQUE I. ESTRUCTURA DEL HOGAR Y PERSONAS CUIDADAS

Sección A. Estructura del hogar
Sección B. Características generales de la persona cuidada

BLOQUE II. CARACTERÍSTICAS E INTENSIDAD DEL CUIDADO

Sección C. Tipo de cuidado (Tareas de cuidado)
Sección D. Tareas específicas relacionadas con el uso de servicios sanitarios para la persona cuidada
Sección E. Frecuencia, intensidad y duración del cuidado
Sección F. Otras cargas de trabajo doméstico y de cuidados de la persona cuidadora
Sección G. Sobrecarga

BLOQUE III. APOYOS, NECESIDADES Y DEMANDAS

Sección H. Apoyo social
Sección I. Apoyo informal para cuidar
Sección J. Apoyo formal para cuidar
Sección K. Necesidades y demandas

BLOQUE IV. ESTADO DE SALUD GENERAL Y MENTAL

Sección L. Estado general de salud
Sección M. Morbilidad, salud mental y consumo de medicamentos
Sección N. Utilización de servicios sanitarios
Sección O. Prácticas preventivas
Sección P. Hábitos saludables
Sección Q. Satisfacción con la vida

BLOQUE V. CONSECUENCIAS DEL CUIDADO EN LA SALUD Y LA CALIDAD DE VIDA

Sección R. Deterioro de la salud
Sección S. Aspectos profesionales y laborales
Sección T. Aspectos económicos
Sección U. Aspectos de ocio, tiempo libre y vida familiar
Sección V. Percepción de efectos positivos del cuidado

BLOQUE VI. VALORACIÓN CONTINGENTE DEL CUIDADO

BLOQUE VII. SOCIODEMOGRÁFICAS

María del Mar García Calvente, María del Río Lozano, Isabel Larrañaga Padilla. Cuestionario del Proyecto CUIDAR-SE: Estudio de seguimiento de la salud y la calidad de vida de mujeres y hombres cuidadores en Andalucía y País Vasco. Granada: Escuela Andaluza de Salud Pública; 2019.
Financiado por el Ministerio de Economía y Competitividad, Instituto de Salud Carlos III y Fondos FEDER (Expte. Nº PI12/00498).
MINISTERIO DE ECONOMÍA Y COMPETITIVIDAD
Instituto de Salud Carlos III
Unión Europea
Fondo Europeo de Desarrollo Regional
"Una manera de hacer Europa"
Andalucía se mueve con Europa

BLOQUE I. ESTRUCTURA DEL HOGAR

SECCIÓN A. ESTRUCTURA DEL HOGAR

A.1. Rellene la siguiente tabla con todos los miembros de su hogar (Instrucciones en página siguiente)

Personas en el hogar	Edad	Sexo	Parentesco de esta persona con usted	Trastorno de salud crónico	Discapacidad	¿Requiere cuidados?	¿Cuál diría usted que es su grado de dependencia?	Grado y nivel de dependencia reconocido	Usted participa en el cuidado	Cuida solo/a o con ayuda	¿Es la persona cuidada a la que va a hacer referencia el cuestionario? [1]
Persona 1											
Persona 2											
Persona 2											
Persona 4											
…											

1. Completar esta columna al final de la sección A, una vez se hallan rellenado las dos tablas. Marcar el número de la persona y poner su nombre. Dirigirse a esta persona en el resto del cuestionario por su nombre.

María del Mar García Calvente, María del Río Lozano, Isabel Larrañaga Padilla. Cuestionario del Proyecto CUIDAR-SE: Estudio de seguimiento de la salud y la calidad de vida de mujeres y hombres cuidadores en Andalucía y País Vasco. Granada: Escuela Andaluza de Salud Pública; 2019.
Financiado por el Ministerio de Economía y Competitividad, Instituto de Salud Carlos III y Fondos FEDER (Expte. Nº PI12/00498).

MINISTERIO DE ECONOMÍA Y COMPETITIVIDAD
Instituto de Salud Carlos III
Unión Europea
Fondo Europeo de Desarrollo Regional
"Una manera de hacer Europa"
Andalucía se mueve con Europa

INSTRUCCIONES PARA TABLA A.1.

Voy a realizarle una serie de preguntas acerca de todos los miembros de su hogar (excluyéndole a usted). Comenzaremos con la persona de mayor edad y terminaremos con la de menor edad de este hogar.

Piense ahora en la Persona 1 (la de mayor edad excluyéndole a usted):
A.1.1.1 (Sección A. Tabla 1. Persona 1. Pregunta 1) ¿Qué edad tiene? _ _ 99 (NS/NC)

A.1.1.2. ¿Es hombre o mujer? H/M

A.1.1.3. Indíqueme cuál es el parentesco de esta persona con usted. Esta persona es su… (mostrar tarjeta)

1. Cónyuge/pareja
2. Hijo/a
3. Padre/madre
4. Suegro/a
5. Otros (familiares)
6. Otros (no familiares)
9. NS/NC

A.1.1.4. ¿Presenta algún trastorno o problema de salud crónico? (se excluye discapacidad)

1. Sí
2. No
9. NS/NC

A.1.1.5. ¿Presenta alguna discapacidad o limitación?

1. Sí
2. No
9. NS/NC

A.1.1.6. Por algún problema de salud crónico, limitación o discapacidad, ¿esta persona no es capaz de cuidarse por sí misma y necesita que le cuide otra persona? (¿Requiere cuidados?)

1. Sí
2. No (Pasar a tabla A.2)
9. NS/NC (Pasar a tabla A.2)

A.1.1.7. En su opinión ¿Cuál diría que es su grado de dependencia? (según su propia percepción, independientemente de que tenga reconocido un grado y nivel)

1. Dependencia moderada (nivel 1: más leve)

Financiado por el Ministerio de Economía y Competitividad, Instituto de Salud Carlos III y Fondos FEDER (Expte. Nº PI12/00498).
MINISTERIO DE ECONOMÍA Y COMPETITIVIDAD
Instituto de Salud Carlos III
Unión Europea
Fondo Europeo de Desarrollo Regional
"Una manera de hacer Europa"
Andalucía se mueve con Europa

2. Dependencia moderada (nivel 2: más dependencia)
3. Dependencia severa (nivel 1: más leve)
4. Dependencia severa (nivel 2: más dependencia)
5. Gran dependencia (nivel 1: más leve)
6. Gran dependencia (nivel 2: más dependencia)
9. NS/NC

A.1.1.8. ¿Tiene algún grado de dependencia reconocido?

1. Sí
2. No (Pasar a A.1.1.10)
9. NS/NC (Pasar a A.1.1.10)

A.1.1.9. ¿Qué grado y nivel de dependencia tiene reconocido?

1. Dependencia moderada (nivel 1: más leve)
2. Dependencia moderada (nivel 2: más dependencia)
3. Dependencia severa (nivel 1: más leve)
4. Dependencia severa (nivel 2: más dependencia)
5. Gran dependencia (nivel 1: más leve)
6. Gran dependencia (nivel 2: más dependencia)
9. NS/NC (Entrevistador/a: en caso de que recuerde o sepa decir el grado pero no el nivel, marcar las dos opciones de ese grado. Es importante que en esta pregunta se pierda la menor información posible)

A.1.1.10. ¿Participa usted en su cuidado?

1. Sí
2. No (Pasar a tabla A.2)
9. NS/NC (Pasar a tabla A.2)

A.1.1.11. ¿Cuida usted solo/a o con ayuda a esta persona?

1. Usted solo/a
2. Usted con su pareja
3. Usted con otra/s persona/s de su hogar (no remunerada)
4. Usted con otra/s persona/s fuera del hogar (no remunerada)
5. Usted con otra/s persona/s remunerada por ello
6. Usted con otro/s profesionales de los servicios sanitarios o sociales
7. Usted con otras personas
9. NS/NC

Entrevistador/a: realizar este mismo bloque de preguntas para cada miembro del hogar (excluyendo a la persona entrevistada).

Aclaraciones sobre el grado y nivel de dependencia para el/la entrevistador/a y para aclarar a la persona entrevistada en caso necesario:

La situación de dependencia se clasificará en los siguientes grados:

María del Mar García Calvente, María del Río Lozano, Isabel Larrañaga Padilla. Cuestionario del Proyecto CUIDAR-SE: Estudio de seguimiento de la salud y la calidad de vida de mujeres y hombres cuidadores en Andalucía y País Vasco. Granada: Escuela Andaluza de Salud Pública; 2019.
Financiado por el Ministerio de Economía y Competitividad, Instituto de Salud Carlos III y Fondos FEDER (Expte. Nº PI12/00498).
MINISTERIO DE ECONOMÍA Y COMPETITIVIDAD
Instituto de Salud Carlos III
Unión Europea
Fondo Europeo de Desarrollo Regional
"Una manera de hacer Europa"
Andalucía se mueve con Europa

a) Grado I. *Dependencia moderada: cuando la persona necesita ayuda para realizar varias actividades básicas de la vida diaria, al menos una vez al día o tiene necesidades de apoyo intermitente o limitado para su autonomía personal.*

b) Grado II. *Dependencia severa: cuando la persona necesita ayuda para realizar varias actividades básicas de la vida diaria dos o tres veces al día, pero no quiere el apoyo permanente de un cuidador o tiene necesidades de apoyo extenso para su autonomía personal.*

c) Grado III. *Gran dependencia: cuando la persona necesita ayuda para realizar varias actividades básicas de la vida diaria varias veces al día y, por su pérdida total de autonomía física, mental, intelectual o sensorial, necesita el apoyo indispensable y continuo de otra persona o tiene necesidades de apoyo generalizado para su autonomía personal.*

Cada uno de los grados de dependencia establecidos en el apartado anterior se clasificarán en dos niveles, en función de la autonomía de las personas y de la intensidad del cuidado que requiere.

A.2. ¿Cuida usted a otras personas que no residen en su hogar?

1. Sí (Rellenar la tabla A.2) (Instrucciones después de la tabla)
2. No (Pasar a pregunta A.3)
9. NS/NC (Pasar a pregunta A.3)

María del Mar García Calvente, María del Río Lozano, Isabel Larrañaga Padilla. Cuestionario del Proyecto CUIDAR-SE: Estudio de seguimiento de la salud y la calidad de vida de mujeres y hombres cuidadores en Andalucía y País Vasco. Granada: Escuela Andaluza de Salud Pública; 2019.
Financiado por el Ministerio de Economía y Competitividad, Instituto de Salud Carlos III y Fondos FEDER (Expte. Nº PI12/00498).
MINISTERIO DE ECONOMÍA Y COMPETITIVIDAD
Instituto de Salud Carlos III
Unión Europea
Fondo Europeo de Desarrollo Regional
"Una manera de hacer Europa"
Andalucía se mueve con Europa

Escuela Andaluza de Salud Pública
CONSEJERÍA DE SALUD Y FAMILIAS

EUSKO JAURLARITZA
GOBIERNO VASCO
OSASUN SAILA
DEPARTAMENTO DE SALUD

TABLA A.2. OTRAS PERSONAS A LAS QUE CUIDA QUE NO RESIDEN EN SU HOGAR

Personas a las que cuida que no residen en el hogar	Edad	Sexo	Parentesco con usted	Trastorno crónico	Discapacidad	¿Cuál diría usted que es su grado de dependencia?	Grado y nivel de dependencia reconocido	¿La cuida sólo/a o con ayuda?	¿Vive en su misma localidad?	¿Es la persona cuidada a la que va a hacer referencia el cuestionario? [1]
Persona 1										
Persona 2										
…										

María del Mar García Calvente, María del Río Lozano, Isabel Larrañaga Padilla. Cuestionario del Proyecto CUIDAR-SE: Estudio de seguimiento de la salud y la calidad de vida de mujeres y hombres cuidadores en Andalucía y País Vasco. Granada: Escuela Andaluza de Salud Pública; 2019.
Financiado por el Ministerio de Economía y Competitividad, Instituto de Salud Carlos III y Fondos FEDER (Expte. Nº PI12/00498).

MINISTERIO DE ECONOMÍA Y COMPETITIVIDAD
Instituto de Salud Carlos III
Unión Europea
Fondo Europeo de Desarrollo Regional
"Una manera de hacer Europa"
Andalucía se mueve con Europa

INSTRUCCIONES PARA TABLA A.2.

Voy a realizarle una serie de preguntas acerca de las personas a las que cuida que no residen en su hogar. Comenzaremos con la persona de mayor edad y terminaremos con la de menor edad.

Piense ahora en la Persona 1 (la de mayor edad):

A.2.1.1. (Edad): Igual que A.1.1.1

A.2.1.2 (Sexo): Igual que A.1.1.2

A.2.1.3 (Parentesco): Igual que A.1.1.3

A.2.1.4 y A.2.1.5 (trastorno crónico y discapacidad): Igual que A.1.1.4 y A.1.15

A.2.1.6, A.2.1.7 y A.2.1.8 (grado y nivel): Igual que A.1.1.7, A.1.18 y A.1.19

A.2.1.9 (con quien cuida): Igual que A.1.1.11

A.2.1.10: ¿Esta persona reside en la misma localidad que usted?

1. Sí
2. No
9. NS/NC

Entrevistador/a: En este momento, vamos a centrarnos ***sólo en una persona cuidada.*** *El/la entrevistador/a seleccionará a esta persona con los siguientes criterios:*
1. La que tenga el mayor grado y nivel de dependencia reconocido en cualquiera de las dos tablas anteriores.
2. Si se da la circunstancia de que haya dos o más personas que cumplen el criterio 1, seleccionar la de mayor edad. En el caso de que haya más de una persona con el mayor grado y nivel de dependencia reconocido, seleccionar la de mayor edad.
3. En el caso de que haya más de una persona que cumpla los dos criterios anteriores, seleccionar la que la persona entrevistada haya percibido con mayor grado y nivel de dependencia.
4. Si sigue habiendo más de una persona que cumple los 3 criterios anteriores, formular a la persona cuidadora A.3 (la que considere que requiere más cuidados de el/ella, teniendo en cuenta el cuidado a nivel global, no sólo a quien dedica más tiempo, sino a quién dedica más esfuerzo, tiempo y responsabilidad).

A.3. ¿Cuál de todas las personas de las que hemos hablado, residan o no en su hogar, considera que requiere más cuidados de su parte? Es decir, ¿a quién dedica más tiempo y esfuerzos en el cuidado?

María del Mar García Calvente, María del Río Lozano, Isabel Larrañaga Padilla. Cuestionario del Proyecto CUIDAR-SE: Estudio de seguimiento de la salud y la calidad de vida de mujeres y hombres cuidadores en Andalucía y País Vasco. Granada: Escuela Andaluza de Salud Pública; 2019.
Financiado por el Ministerio de Economía y Competitividad, Instituto de Salud Carlos III y Fondos FEDER (Expte. Nº PI12/00498).

MINISTERIO DE ECONOMÍA Y COMPETITIVIDAD
Instituto de Salud Carlos III
Unión Europea
Fondo Europeo de Desarrollo Regional
"Una manera de hacer Europa"
Andalucía se mueve con Europa

Marcar la opción en la tabla correspondiente, anotando su nombre (X). A partir de aquí nos vamos a referir sólo al cuidado de esta persona, a la que nos referiremos por su nombre cuando aparezca X.

Advertir a la persona entrevistada: A partir de aquí nos vamos a centrar sólo en la persona a la que cuida de forma principal, X. Le pido que piense sólo en el cuidado que realiza con esta persona.

SECCIÓN B. CARACTERÍSTICAS GENERALES DE LA PERSONA CUIDADA

B.1. En relación con otras personas de su edad, ¿cómo diría usted que es el estado de salud de X (nombre)?

1. Muy bueno
2. Bueno
3. Regular
4. Malo
5. Muy malo
9. NS/NC

B.2. Pensando ahora en los últimos 6 meses, ¿en qué medida se ha visto limitado/a X debido a un problema de salud para realizar las actividades que la gente habitualmente hace?

1. Gravemente limitado/a
2. Limitado/a, pero no gravemente
3. Nada limitado/a
9. NS/NC

B.3. ¿Qué tipo de problema es la causa de su dificultad para realizar las actividades que la gente habitualmente hace?

1. Físico (pasar a B.5)
2. Mental
3. Ambos
9. NS/NC (pasar a B.5)

B.4. ¿Presenta deterioro cognitivo (tipo alzheimer o demencias)?

1. Sí
2. No
9. NS/NC

B.5. ¿Cuál es el problema/condición/enfermedad que generó la situación de dependencia en X?

1. Congénito (problema anterior al parto)

María del Mar García Calvente, María del Río Lozano, Isabel Larrañaga Padilla. Cuestionario del Proyecto CUIDAR-SE: Estudio de seguimiento de la salud y la calidad de vida de mujeres y hombres cuidadores en Andalucía y País Vasco. Granada: Escuela Andaluza de Salud Pública; 2019.
Financiado por el Ministerio de Economía y Competitividad, Instituto de Salud Carlos III y Fondos FEDER (Expte. Nº PI12/00498).

MINISTERIO DE ECONOMÍA Y COMPETITIVIDAD
Instituto de Salud Carlos III
Unión Europea
Fondo Europeo de Desarrollo Regional
"Una manera de hacer Europa"
Andalucía se mueve con Europa

2. Problemas en el parto
2. Un accidente
3. Una enfermedad
4. Otras causas ¿Cuál?
9. NS/NC

B.6. ¿Tiene X alguna dificultad de las que le cito a continuación?

1. Visión
2. Audición
3. Comunicación
4. Aprendizaje y aplicación del conocimiento y desarrollo de tareas
5. Movilidad
6. Autocuidado
7. Tareas de la vida doméstica
8. Relaciones interpersonales (comportamentales)

B.7. ¿Cuánto tiempo hace que X tiene el impedimento/limitación/dificultad que generó su situación de dependencia?

Meses: (contestar en meses sólo si hace menos de 24 meses)
Años:
NS/NC

B.8. En relación con la salud de X, piensa que cada día que pasa está…

1. Mejor
2. Peor
3. No cambia
9. NS/NC

María del Mar García Calvente, María del Río Lozano, Isabel Larrañaga Padilla. Cuestionario del Proyecto CUIDAR-SE: Estudio de seguimiento de la salud y la calidad de vida de mujeres y hombres cuidadores en Andalucía y País Vasco. Granada: Escuela Andaluza de Salud Pública; 2019.
Financiado por el Ministerio de Economía y Competitividad, Instituto de Salud Carlos III y Fondos FEDER (Expte. Nº PI12/00498).
MINISTERIO DE ECONOMÍA Y COMPETITIVIDAD
Instituto de Salud Carlos III
Unión Europea
Fondo Europeo de Desarrollo Regional
"Una manera de hacer Europa"
Andalucía se mueve con Europa

BLOQUE II. CARACTERÍSTICAS E INTENSIDAD DEL CUIDADO

SECCIÓN C. TAREAS DE CUIDADO (EN GENERAL)

C.1. ¿Realiza usted las siguientes tareas que le voy a mencionar a continuación como parte del cuidado de X?
Entrevistador/a: En la tabla C, marque una x en todas las tareas que realiza. Para los grupos de tareas en los que ha marcado al menos una x, realizar la siguiente pregunta (¿Quién la realiza?)

Entrevistador/a: Poner una x si participa en la realización de esta tarea. Realizar primero esta columna para todas las tareas. Una vez que esté rellena esta columna, preguntar quién hace cada grupo de tareas (agrupadas en lugar de forma individual), en aquellos grupos en los que hay al menos una tarea en la que ha contestado que sí realiza.

C.2. ¿Quién realiza este tipo de tareas relacionadas con… [Mencionar sólo los grupos de tareas en los que se haya marcado una x en, al menos, una tarea].

María del Mar García Calvente, María del Río Lozano, Isabel Larrañaga Padilla. Cuestionario del Proyecto CUIDAR-SE: Estudio de seguimiento de la salud y la calidad de vida de mujeres y hombres cuidadores en Andalucía y País Vasco. Granada: Escuela Andaluza de Salud Pública; 2019.
Financiado por el Ministerio de Economía y Competitividad, Instituto de Salud Carlos III y Fondos FEDER (Expte. Nº PI12/00498).

MINISTERIO DE ECONOMÍA Y COMPETITIVIDAD
Instituto de Salud Carlos III
Unión Europea
Fondo Europeo de Desarrollo Regional
"Una manera de hacer Europa"
Andalucía se mueve con Europa

TABLA C. TAREAS

Grupos de tareas	Tareas: Ayudarle a…	¿Realiza?	¿Quién realiza este tipo de tareas?
Cuidados personales	Dar la comida, ayudar a comer		1. Usted solo/a<br>2. Usted junto con otra/s persona/s del hogar (no remuneradas)<br>3. Usted junto con otra/s persona/s de fuera del hogar (no remuneradas)<br>4. Usted junto con otra/s persona/s remunerada/s por ello<br>5. Usted con otros profesional/es de los servicios sociales<br>6. Usted con otras personas<br>9. NS/NC
	Vestirse y desvestirse		
	Abrocharse los zapatos		
	Bañarse o ducharse		
	Lavarse o asearse		
	Ayuda para ir al retrete		
	Cambio de pañales (incontinencia de orina)		
	Cambio de pañales (incontinencia fecal)		
Movilidad física	Acostarse/levantarse de la cama		1 2 3 4 5 6 9<br>(Igual que arriba)
	Andar o desplazarse por la casa		
	Subir o bajar escaleras		
Tareas domésticas o de acompañamiento dentro del hogar	Preparar la comida (para la persona cuidada)		1 2 3 4 5 6 9
	Realizar otras tareas domésticas (extras) relacionadas con el cuidado (poner lavadoras, tareas de limpieza…)		
	Acompañar o vigilar a la persona cuidada (dentro del hogar)		
Cuidados relacionados con la atención a la enfermedad	Administrar medicación o vigilar para que la tome		1 2 3 4 5 6 9
	Realizar curas, poner sondas, movilizar la cama		
	Prestar otro tipo de cuidados, como fisioterapia, rehabilitación o estimulación		
Utilización de servicios sanitarios	Hacer gestiones médicas para la persona cuidada		1 2 3 4 5 6 9
	Ir al médico/a		
Tareas fuera del hogar	Salir a la calle /desplazarse por la calle		1 2 3 4 5 6 9
	Ayudar a utilizar el transporte público		
	Hacer compras		
	Hacer gestiones administrativas		

María del Mar García Calvente, María del Río Lozano, Isabel Larrañaga Padilla. Cuestionario del Proyecto CUIDAR-SE: Estudio de seguimiento de la salud y la calidad de vida de mujeres y hombres cuidadores en Andalucía y País Vasco. Granada: Escuela Andaluza de Salud Pública; 2019.
Financiado por el Ministerio de Economía y Competitividad, Instituto de Salud Carlos III y Fondos FEDER (Expte. Nº PI12/00498).

SECCIÓN D. TAREAS DE CUIDADO ESPECÍFICAS RELACIONADAS CON EL USO DE SERVICIOS SANITARIOS

D.1. En las últimas cuatro semanas, ¿ha consultado usted a algún médico de familia/ médico general o médico especialista por algún problema, molestia o enfermedad de X, acompañando o sin acompañar a X? (No se incluye hospitalización).

1. Sí ----------------D.2. ¿Cuántas veces?
2. No
9. NS/NC

D.3. Durante los últimos 12 meses, ¿ha estado X hospitalizada como paciente al menos durante una noche?

1. Sí ----------------D.4. ¿Cuántas veces?
2. No (Pasar a D.9)
9. NS/NC (Pasar a D.9)

D.5. Refiriéndonos ahora al último ingreso ¿Cuántos días estuvo ingresado/a?

Nº de días: _ _

D.6 Durante su último ingreso, ¿le acompañó usted en el hospital?

1. Sí
2. No (pasar a D.9)
9. NS/NC (pasar a D.9)

D.7. ¿Con qué frecuencia le acompañó en su último ingreso?

1. Todos los días
2. Algunos días
3. De vez en cuando
4. NS/NC (pasar a D.9)

D.8. Cuando le acompañó en su último ingreso…

1. Se quedaba durante todo el día, incluyendo la noche
2. Se quedaba durante todo el día, excluyendo la noche
3. Se quedaba durante toda la noche (no durante el día)
4. Se quedaba algunas horas durante el día
5. Se quedaba algunas horas durante la noche
5. Otros
9. NS/NC

D.9. Durante los últimos 12 meses, ¿ha tenido que acompañarle usted a X a algún servicio de urgencias?

1. Sí -------------------D.10 ¿Cuántas veces?

María del Mar García Calvente, María del Río Lozano, Isabel Larrañaga Padilla. Cuestionario del Proyecto CUIDAR-SE: Estudio de seguimiento de la salud y la calidad de vida de mujeres y hombres cuidadores en Andalucía y País Vasco. Granada: Escuela Andaluza de Salud Pública; 2019.
Financiado por el Ministerio de Economía y Competitividad, Instituto de Salud Carlos III y Fondos FEDER (Expte. Nº PI12/00498).
MINISTERIO DE ECONOMÍA Y COMPETITIVIDAD
Instituto de Salud Carlos III
Unión Europea
Fondo Europeo de Desarrollo Regional
"Una manera de hacer Europa"
Andalucía se mueve con Europa

2. No
9. NS/NC

D.11. Durante los últimos 12 meses, ¿ha tenido que acompañarle usted a X a otros servicios sanitarios, como enfermería, rehabilitación o fisioterapia?
1. Sí -------------------D.12 ¿Cuántas veces?
2. No
9. NS/NC

SECCIÓN E. FRECUENCIA, INTENSIDAD Y DURACIÓN DEL CUIDADO

E.1. ¿Cuántos días a la semana dedica a cuidar a X?
1. Uno
2. Dos
3. Tres
4. Cuatro
5. Cinco
6. Seis
7. Siete
9. NS/NC

E.2. Pensando en todas las atenciones que presta a X ¿cuántas horas le dedica usted, como media, en un día de diario (de lunes a viernes)?
De lunes a viernes (promedio diario) Nº horas: _ _ NS/NC _ (99)

E.3. ¿Qué promedio de horas le dedica usted en un sábado?
Nº horas: _ _ NS/NC_ (99)

E.4. ¿Y qué promedio de horas le dedica en un domingo?
Nº horas: _ _ NS/NC_ (99)

E.4. ¿Cuánto tiempo hace que cuida usted a esta persona?
Nº años:_ _ (En caso de que sea inferior a un año, poner meses: _ _) NS/NC _ (99)

E.5. ¿Cree que ahora dedica más o menos tiempo al cuidado que cuando comenzó a cuidar a esta persona?
1. Mucho más
2. Más
2. Igual
3. Menos
4. Mucho menos
9. NS/NC

Financiado por el Ministerio de Economía y Competitividad, Instituto de Salud Carlos III y Fondos FEDER (Expte. Nº PI12/00498).
MINISTERIO DE ECONOMÍA Y COMPETITIVIDAD
Instituto de Salud Carlos III
Unión Europea
Fondo Europeo de Desarrollo Regional
"Una manera de hacer Europa"
Andalucía se mueve con Europa

SECCIÓN F. OTRAS CARGAS DE TRABAJO DOMÉSTICO Y DE CUIDADOS DE LA PERSONA CUIDADORA

Entrevistador/a: ver tablas A.1 y A.2. (Sección A). Si cuida a más de una persona, formular F.1. Si no, pasar a F.2.

F.1. En total, ¿cuántos días a la semana cuida, contando a todas las personas a las que cuida, ya residan en este o en otro hogar?
1. Uno
2. Dos
3. Tres
4. Cuatro
5. Cinco
6. Seis
7. Siete
9. NS/NC

Entrevistador/a: ver tablas A.1 y A.2. (Sección A). Si hay en el hogar al menos una persona menor de 16 años (15 o menos), formular F.2. Si no, pasar a F.3.

F.2. ¿Quién se ocupa principalmente del cuidado de la/s persona/s menor/es de 16 años de este hogar?
1. Vd. solo/a
2. Vd. compartiéndolo con otra persona. Indique cuál ____ (mostrar tarjeta que se utilizará para J.4 y J.7, ver sección J)
3. Otra persona de la casa. Indique cuál _____ (mostrar tarjeta de sección J)
4. Una persona que NO reside en el hogar remunerada por ello
5. Los servicios sociales
6. Otra situación ----- Especifique cuál
9. NS/NC

F.3. ¿Quién se ocupa principalmente de las tareas domésticas de su hogar?
1. Vd. solo/a
2. Vd. compartiéndolo con otra persona. Indique cuál
3. Otra persona de la casa. Indique cuál.
4. Una persona que NO reside en el hogar remunerada por ello
5. Los servicios sociales
6. Otra situación -----------Especifique cuál
9. NS/NC

F.4. ¿Cuántas horas dedica usted a las tareas domésticas?
De lunes a viernes (promedio diario) Nº horas: _ _ NS/NC _ (99)
Sábado Nº horas: _ _ NS/NC _ (99)
Domingo Nº horas: _ _ NS/NC _ (99)

María del Mar García Calvente, María del Río Lozano, Isabel Larrañaga Padilla. Cuestionario del Proyecto CUIDAR-SE: Estudio de seguimiento de la salud y la calidad de vida de mujeres y hombres cuidadores en Andalucía y País Vasco. Granada: Escuela Andaluza de Salud Pública; 2019.
Financiado por el Ministerio de Economía y Competitividad, Instituto de Salud Carlos III y Fondos FEDER (Expte. Nº PI12/00498).
MINISTERIO DE ECONOMÍA Y COMPETITIVIDAD
Instituto de Salud Carlos III
Unión Europea
Fondo Europeo de Desarrollo Regional
"Una manera de hacer Europa"
Andalucía se mueve con Europa

F.5. ¿Cuenta con alguna persona remunerada para las tareas domésticas?
1. Sí
2. No
9. NS/NC

SECCIÓN G. SOBRECARGA (ESCALA ZARIT)[1]

Las preguntas que siguen se refieren a cómo se siente y a lo que le ocurre. En cada pregunta responda lo que más se aproxime a cómo se siente usted.
Instrucciones. Después de leer cada frase, indique con qué frecuencia se siente usted de esa manera, escogiendo entre nunca, casi nunca, a veces, frecuentemente o casi siempre. No existen respuestas correctas o incorrectas. El resultado es la suma de las puntuaciones.

1. ¿Siente usted que su familiar solicita más ayuda de la que realmente necesita?

Nunca	Casi nunca	A veces	Frecuentemente	Casi siempre
1	2	3	4	5

2. ¿Siente usted que, a causa del tiempo que gasta con su familiar, ya no tiene tiempo suficiente para usted mismo?

Nunca	Casi nunca	A veces	Frecuentemente	Casi siempre
1	2	3	4	5

3. ¿Se siente estresada(o) al tener que cuidar a su familiar y tener además que atender otras responsabilidades? (P. ej., con su familia o en el trabajo)

Nunca	Casi nunca	A veces	Frecuentemente	Casi siempre
1	2	3	4	5

4. ¿Se siente avergonzada(o) por el comportamiento de su familiar?

Nunca	Casi nunca	A veces	Frecuentemente	Casi siempre
1	2	3	4	5

5. ¿Se siente irritada(o) cuando está cerca de su familiar?

Nunca	Casi nunca	A veces	Frecuentemente	Casi siempre
1	2	3	4	5

6. ¿Cree que la situación actual afecta a su relación con amigos u otros miembros de su familia de una forma negativa?

Nunca	Casi nunca	A veces	Frecuentemente	Casi siempre
1	2	3	4	5

7. ¿Siente temor por el futuro que le espera a su familiar?

Nunca	Casi nunca	A veces	Frecuentemente	Casi siempre
1	2	3	4	5

---

[1] Escala de Zarit validada en español.
Martín M, Salvadó I, Nadal S, Miji LC, Rico JM, Lanz P, et al. Adaptación para nuestro medio de la Escala de Sobrecarga del Cuidador (Caregiver Burden Interview) de Zarit. Rev Gerontol. 1996;6:338-46.

Financiado por el Ministerio de Economía y Competitividad, Instituto de Salud Carlos III y Fondos FEDER (Expte. Nº PI12/00498).

8. ¿Siente que su familiar depende de usted?

Nunca	Casi nunca	A veces	Frecuentemente	Casi siempre
1	2	3	4	5

9. ¿Se siente agotada(o) cuando tiene que estar junto a su familiar?

Nunca	Casi nunca	A veces	Frecuentemente	Casi siempre
1	2	3	4	5

10. ¿Siente usted que su salud se ha visto afectada por tener que cuidar a su familiar?

Nunca	Casi nunca	A veces	Frecuentemente	Casi siempre
1	2	3	4	5

11. ¿Siente que no tiene la vida privada que desearía a causa de su familiar?

Nunca	Casi nunca	A veces	Frecuentemente	Casi siempre
1	2	3	4	5

12. ¿Cree que sus relaciones sociales se han visto afectadas por tener que cuidar a su familiar?

Nunca	Casi nunca	A veces	Frecuentemente	Casi siempre
1	2	3	4	5

13. (Solamente si el/la entrevistado/a vive con la persona a la que cuida.) ¿Se siente incómoda(o) para invitar amigos a su casa, a causa de su familiar?

Nunca	Casi nunca	A veces	Frecuentemente	Casi siempre
1	2	3	4	5

14. ¿Cree que su familiar espera que usted le cuide como si fuera la única persona con la que pudiera contar?

Nunca	Casi nunca	A veces	Frecuentemente	Casi siempre
1	2	3	4	5

15. ¿Cree usted que no dispone de dinero suficiente para cuidar de su familiar, además de sus otros gastos?

Nunca	Casi nunca	A veces	Frecuentemente	Casi siempre
1	2	3	4	5

16. ¿Siente que no va a ser capaz de cuidar a su familiar durante mucho más tiempo?

Nunca	Casi nunca	A veces	Frecuentemente	Casi siempre
1	2	3	4	5

17. ¿Siente que ha perdido el control sobre su vida desde que la enfermedad de su familiar se manifestó?

Nunca	Casi nunca	A veces	Frecuentemente	Casi siempre
1	2	3	4	5

18. ¿Desearía poder encargar el cuidado de su familiar a otra persona?

Nunca	Casi nunca	A veces	Frecuentemente	Casi siempre
1	2	3	4	5

19. ¿Se siente insegura(o) acerca de lo que debe hacer con su familiar?

Nunca	Casi nunca	A veces	Frecuentemente	Casi siempre
1	2	3	4	5

María del Mar García Calvente, María del Río Lozano, Isabel Larrañaga Padilla. Cuestionario del Proyecto CUIDAR-SE: Estudio de seguimiento de la salud y la calidad de vida de mujeres y hombres cuidadores en Andalucía y País Vasco. Granada: Escuela Andaluza de Salud Pública; 2019.
Financiado por el Ministerio de Economía y Competitividad, Instituto de Salud Carlos III y Fondos FEDER (Expte. Nº PI12/00498).

20. ¿Siente que debería hacer más de lo que hace por su familiar?

Nunca	Casi nunca	A veces	Frecuentemente	Casi siempre
1	2	3	4	5

21. ¿Cree que podría cuidar a su familiar mejor de lo que lo hace?

Nunca	Casi nunca	A veces	Frecuentemente	Casi siempre
1	2	3	4	5

22. En general, ¿se siente muy sobrecargada(o) al tener que cuidar de su familiar?

Nunca	Casi nunca	A veces	Frecuentemente	Casi siempre
1	2	3	4	5

Financiado por el Ministerio de Economía y Competitividad, Instituto de Salud Carlos III y Fondos FEDER (Expte. Nº PI12/00498).
MINISTERIO DE ECONOMÍA Y COMPETITIVIDAD
Instituto de Salud Carlos III
Unión Europea
Fondo Europeo de Desarrollo Regional
"Una manera de hacer Europa"
Andalucía se mueve con Europa

BLOQUE III. APOYOS, NECESIDADES Y DEMANDAS

SECCIÓN H. APOYO SOCIAL (ESCALA DUKE-11)[2]

H.1. Voy a entregarle una tarjeta con diferentes situaciones de apoyo afectivo y personal que suelen ocurrir en la vida cotidiana. Al lado de cada situación hay un conjunto de respuestas. Por favor, lea cada situación y dígame una por una el número de la respuesta que mejor refleje su situación.

Entrevistador/a: Entregue la tarjeta. A continuación diga: "Para la situación 1, dígame, por favor, el número de su respuesta". Después de anotar el número, proceda de la misma forma hasta terminar con todas las situaciones.

	Mucho menos de lo que deseo	Menos de lo que deseo	Ni mucho ni poco	Casi como deseo	Tanto como deseo	NS	NC
1. Recibo visitas de mis amigos/as y familiares	1	2	3	4	5	8	9
2. Recibo ayuda en asuntos relacionados con mi casa	1	2	3	4	5	8	9
3. Recibo elogios y reconocimientos cuando hago bien mi trabajo	1	2	3	4	5	8	9
4. Cuento con personas que se preocupan de lo que me sucede	1	2	3	4	5	8	9
5. Recibo amor y afecto	1	2	3	4	5	8	9
6. Tengo la posibilidad de hablar con alguien de mis problemas en el trabajo o en la casa	1	2	3	4	5	8	9
7. Tengo la posibilidad de hablar con alguien de mis problemas personales y familiares	1	2	3	4	5	8	9
8. Tengo la posibilidad de hablar con alguien de mis problemas económicos	1	2	3	4	5	8	9
9. Recibo invitaciones para distraerme y salir con otras personas	1	2	3	4	5	8	9
10. Recibo consejos útiles cuando me ocurre algún acontecimiento importante en mi vida	1	2	3	4	5	8	9
11. Recibo ayuda cuando estoy enfermo/a en la cama	1	2	3	4	5	8	9

[2] JA Bellón Saameño, A Delgado Sánchez, J de Dios Luna del Castillo, P Lardelli Claret (1996).Validez y fiabilidad del cuestionario de apoyo social funcional Duke-UNC-11. Atención Primaria 18 (4).

Financiado por el Ministerio de Economía y Competitividad, Instituto de Salud Carlos III y Fondos FEDER (Expte. Nº PI12/00498).

MINISTERIO DE ECONOMÍA Y COMPETITIVIDAD
Instituto de Salud Carlos III
Unión Europea
Fondo Europeo de Desarrollo Regional
"Una manera de hacer Europa"
Andalucía se mueve con Europa

SECCIÓN I. APOYO INFORMAL PARA CUIDAR

I.1. Voy a leerle una relación de personas que pueden pertenecer al entorno familiar o no. Dígame en qué grado (mucho, bastante, poco o nada) le ayudan en la actualidad esas personas en el cuidado de X.

	No existe esa persona	Grado de ayuda				
		Mucho	Bastante	Algo	Nada	NS/NC
1. Su esposa o pareja (Mujer)	0	4	3	2	1	9
2. Su esposo o pareja (Hombre)	0	4	3	2	1	9
3. Su hija (M)	0	4	3	2	1	9
4. Su hijo (H)	0	4	3	2	1	9
5. Su madre (M)	0	4	3	2	1	9
6. Su padre (H)	0	4	3	2	1	9
7. Su hermana (M)	0	4	3	2	1	9
8. Su hermano (H)	0	4	3	2	1	9
9. Su suegra (M)	0	4	3	2	1	9
10. Su suegro (H)	0	4	3	2	1	9
11. Otros familiares (Indique cuál--)	0	4	3	2	1	9
12. Otras personas no familiares (amigos/as, vecinos/as…)	0	4	3	2	1	9
13. Otra persona remunerada	0	4	3	2	1	9

I.2. ¿Cuántas personas en total participan en el cuidado de X?

Número de personas: _ _ NS/NC _ 99

I.3. Teniendo en cuenta todo tipo de apoyo, ¿hay alguna persona que le ayude especialmente a atender a X?

1. Sí
2. No (pasar a J.5)
9. NC (pasar a J.5)

I.4. ¿Quién es esa persona? (Mostrar tarjeta con clasificación de 13 categorías de la tabla J.1?

1. Su esposa o pareja (Mujer)
2. Su esposo o pareja (Hombre)
3. Su hija (M)
4. Su hijo (H)
5. Su madre (M)
6. Su padre (H)
7. Su hermana (M)
8. Su hermano (H)
9. Su suegra (M)
10. Su suegro (H)
11. Otros familiares (Indique cuál--)

Financiado por el Ministerio de Economía y Competitividad, Instituto de Salud Carlos III y Fondos FEDER (Expte. Nº PI12/00498).
MINISTERIO DE ECONOMÍA Y COMPETITIVIDAD
Instituto de Salud Carlos III
Unión Europea
Fondo Europeo de Desarrollo Regional
"Una manera de hacer Europa"
Andalucía se mueve con Europa

12. Otras personas no familiares (amigos/as, vecinos/as…)
13. Otra persona remunerada
99. NS/NC

I.5. ¿En qué medida considera que necesita apoyo para cuidar a X?
1. No necesita apoyo (pasar a J.7)
2. Necesita poco apoyo
3. Necesita bastante apoyo
4. Necesita mucho apoyo
9. NS/NC

I.6. ¿Pide ayuda para cuidar cuando la necesita?
1. Sí, siempre que la necesita.
2. Casi siempre que la necesita
3. Algunas veces, pero menos de lo que necesita
4. No suele hacerlo casi nunca, aunque lo necesite
5. Nunca pide ayuda
9. NS/NC

I.7. Cuando usted necesita ayuda para atender a X. ¿A quién acude en primer lugar? (mostrar tarjeta)
1. Su esposa o pareja (Mujer)
2. Su esposo o pareja (Hombre)
3. Su hija (M)
4. Su hijo (H)
5. Su madre (M)
6. Su padre (H)
7. Su hermana (M)
8. Su hermano (H)
9. Su suegra (M)
10. Su suegro (H)
11. Otros familiares (Indique cuál--)
12. Otras personas no familiares (amigos/as, vecinos/as…)
13. Otra persona remunerada
99. NS/NC

I.8. En la actualidad, cuando necesita ayuda de algún familiar, amigo/a o vecino/a, la obtiene…
1. Siempre
2. Casi siempre
3. Algunas veces
4. Sólo alguna vez
5. Nunca
9. NC

María del Mar García Calvente, María del Río Lozano, Isabel Larrañaga Padilla. Cuestionario del Proyecto CUIDAR-SE: Estudio de seguimiento de la salud y la calidad de vida de mujeres y hombres cuidadores en Andalucía y País Vasco. Granada: Escuela Andaluza de Salud Pública; 2019.
Financiado por el Ministerio de Economía y Competitividad, Instituto de Salud Carlos III y Fondos FEDER (Expte. Nº PI12/00498).
MINISTERIO DE ECONOMÍA Y COMPETITIVIDAD
Instituto de Salud Carlos III
Unión Europea
Fondo Europeo de Desarrollo Regional
"Una manera de hacer Europa"
Andalucía se mueve con Europa

SECCIÓN J. APOYO FORMAL PARA CUIDAR

J.1. Por favor, dígame si conoce la existencia de los siguientes servicios que le voy a leer.
Entrevistador/a: Leer servicios y prestaciones y anotar los que conoce en la columna J.1 de la tabla siguiente.
Si no conoce ninguno, fin de la tabla (pasar a J.7)
Para todos los servicios que conoce, formular J.2 y J.3.

J.2. ¿En qué medida (mucho, bastante, poco o nada) considera que este tipo de servicios o prestaciones es de utilidad para usted?
Entrevistador/a: Anote en la columna J.2. de la tabla

J.3. ¿Cuáles de estos servicios o prestaciones ha utilizado usted alguna vez?
Entrevistador/a: Anote en la columna J.3 de la tabla.
Si no ha utilizado ninguno, fin de tabla (pasar a J.7)
Para todos los servicios o prestaciones que ha utilizado, formular J.4, J.5 y J.6.

J.4. Durante los últimos doce meses, ¿cuántas veces ha utilizado este servicio?
Entrevistador/a: Anote el número en la columna J.4 de la tabla y pase a J.5.

J.5. Y el servicio que ha utilizado, ¿era un servicio público, un servicio privado, dependía de una organización de voluntariado o ha utilizado una combinación de varios tipos de servicios?
Entrevistador/a: Anote en la columna J.5 de la tabla y pase a J.6.

K.6. ¿En qué medida (mucho, bastante, poco o nada) está satisfecho/a con este tipo de servicio que ha recibido?
Entrevistador/a: Anote en la columna J.6 de la tabla.

María del Mar García Calvente, María del Río Lozano, Isabel Larrañaga Padilla. Cuestionario del Proyecto CUIDAR-SE: Estudio de seguimiento de la salud y la calidad de vida de mujeres y hombres cuidadores en Andalucía y País Vasco. Granada: Escuela Andaluza de Salud Pública; 2019.
Financiado por el Ministerio de Economía y Competitividad, Instituto de Salud Carlos III y Fondos FEDER (Expte. Nº PI12/00498).
MINISTERIO DE ECONOMÍA Y COMPETITIVIDAD
Instituto de Salud Carlos III
Unión Europea
Fondo Europeo de Desarrollo Regional
"Una manera de hacer Europa"
Andalucía se mueve con Europa

Servicios sociales y sanitarios	J.1			J.2					J.3			J.4	J.5					J.6				
	Conoce			Utilidad					¿Ha utilizado?			Nº veces	Tipo de servicio					Satisfacción				
	Sí	No	NS/C	M	B	P	N	NS/C	Si	No	NS/C		Púb.	Priv	Vol.	Mix.	NS/C	M	B	P	N	NS/C
(1) Prestación económica para cuidados en el entorno familiar	1	2	9	1	2	3	4	9	1	2	3		1	2	3	4	9	1	2	3	4	9
(2) Otras prestaciones económicas	1	2	9	1	2	3	4	9	1	2	3		1	2	3	4	9	1	2	3	4	9
(3) Servicio de Teleasistencia	1	2	9	1	2	3	4	9	1	2	3		1	2	3	4	9	1	2	3	4	9
(4) Servicio de Ayuda a Domicilio	1	2	9	1	2	3	4	9	1	2	3		1	2	3	4	9	1	2	3	4	9
(5.1) Servicio de Centro de Día	1	2	9	1	2	3	4	9	1	2	3		1	2	3	4	9	1	2	3	4	9
(5.2) Servicio de Centro de Noche	1	2	9	1	2	3	4	9	1	2	3		1	2	3	4	9	1	2	3	4	9
(6.1) Servicio de Atención Residencial (respiro temporal)	1	2	9	1	2	3	4	9	1	2	3		1	2	3	4	9	1	2	3	4	9
(6.2) Servicio de Atención Residencial (permanente)	1	2	9	1	2	3	4	9	1	2	3		1	2	3	4	9	1	2	3	4	9
(7) Información y orientación	1	2	9	1	2	3	4	9	1	2	3		1	2	3	4	9	1	2	3	4	9
(8) Cursos o talleres de formación	1	2	9	1	2	3	4	9	1	2	3		1	2	3	4	9	1	2	3	4	9
(9) Grupos de apoyo o ayuda mutua	1	2	9	1	2	3	4	9	1	2	3		1	2	3	4	9	1	2	3	4	9
(10) Apoyo psicológico o consejo emocional	1	2	9	1	2	3	4	9	1	2	3		1	2	3	4	9	1	2	3	4	9

María del Mar García Calvente, María del Río Lozano, Isabel Larrañaga Padilla. Cuestionario del Proyecto CUIDAR-SE: Estudio de seguimiento de la salud y la calidad de vida de mujeres y hombres cuidadores en Andalucía y País Vasco. Granada: Escuela Andaluza de Salud Pública; 2019.
Financiado por el Ministerio de Economía y Competitividad, Instituto de Salud Carlos III y Fondos FEDER (Expte. Nº PI12/00498).

(11) Servicio de enfermería a domicilio	1	2	9	1	2	3	4	9	1	2	3		1	2	3	4	9	1	2	3	4	9
(12) Enfermeras/os gestoras/es de casos	1	2	9	1	2	3	4	9	1	2	3		1	2	3	4	9	1	2	3	4	9
(13) Trabajadores/as o asistentes sociales	1	2	9	1	2	3	4	9	1	2	3		1	2	3	4	9	1	2	3	4	9
(14) Servicio de comida	1	2	9	1	2	3	4	9	1	2	3		1	2	3	4	9	1	2	3	4	9
(15) Servicio de transporte	1	2	9	1	2	3	4	9	1	2	3		1	2	3	4	9	1	2	3	4	9

María del Mar García Calvente, María del Río Lozano, Isabel Larrañaga Padilla. Cuestionario del Proyecto CUIDAR-SE: Estudio de seguimiento de la salud y la calidad de vida de mujeres y hombres cuidadores en Andalucía y País Vasco. Granada: Escuela Andaluza de Salud Pública; 2019.
Financiado por el Ministerio de Economía y Competitividad, Instituto de Salud Carlos III y Fondos FEDER (Expte. Nº PI12/00498).

Definiciones de los servicios y prestaciones para el entrevistador/a: (Clarifique sólo si es necesario)

1. ***Prestación económica para cuidados en el entorno familiar:*** *Su finalidad es mantener al/la beneficiario/a en su domicilio atendido por cuidadores/as no profesionales, siempre que se den condiciones adecuadas de convivencia y de habitabilidad de la vivienda.*

2. ***Otras prestaciones económicas****:*

    a. ***Prestación económica de asistencia personal:*** *Destinada a facilitar la promoción de la autonomía de la persona con gran dependencia, con independencia de su edad. Contribuye a la contratación de una asistencia personal que facilite al/la beneficiario/a una vida más autónoma, el acceso a la educación y al trabajo y el ejercicio de las Actividades básicas de la vida diaria (ABVD).*

    b. ***Prestación económica vinculada al servicio:*** *Prestación personal y periódica destinada a la cobertura de los gastos del servicio previsto en el Programa Individual de Atención (PIA) cuando no sea posible la atención por un servicio público o concertado de atención y cuidado.*

    c. ***Otro tipo de prestaciones económicas***

3. ***Servicio de Teleasistencia:*** *atiende a los/as beneficiario/as mediante el uso de tecnologías de la comunicación y apoyo de medios personales con objeto de dar respuesta inmediata en situaciones de emergencia o de inseguridad, soledad y aislamiento. Se incluyen aquí otros dispositivos a distancia similares para ayudar a la persona cuidadora en sus labores de cuidado.*

4. ***Ayuda a Domicilio****: La Ayuda a Domicilio comprende la atención personal en la realización de las actividades de la vida diaria y la cobertura de las necesidades domésticas mediante los servicios previstos en la ley de dependencia y los que en su desarrollo puedan establecerse por las correspondientes Comunidades Autónomas o Administración que, en su caso, tenga la competencia.*

5. ***Centro de Día y de Noche****: El Centro de Día y de Noche ajusta los servicios establecidos en la ley de dependencia, a las necesidades de las personas en situación de dependencia atendidas según su grado y nivel. La intensidad de este servicio estará en función de los servicios del centro que precisa la persona en situación de dependencia, de acuerdo con su PIA.*

6. ***Servicio de Atención Residencial****: Este servicio ofrece una atención integral y continuada de carácter personal, social y sanitario, que se prestará en centros residenciales, públicos o acreditados. Puede tener carácter* **permanente o temporal***, con motivo de vacaciones, fines de semana, convalecencia, enfermedades o descansos de los cuidadores no profesionales. El servicio de atención residencial ajustará los servicios y programas de intervención a las necesidades de las personas en situación de dependencia atendidas; así mismo su intensidad estará en función de los servicios que precisa la persona en situación de dependencia, de acuerdo con su PIA.*

7. ***Actuaciones de información y orientación****: Información y orientación sobre la enfermedad de la persona dependiente, sus consecuencias y el manejo de los problemas asociados a su cuidado, sobre las dificultades más comunes y sus posibles soluciones, estrategias y recursos para hacerles frente.*

Financiado por el Ministerio de Economía y Competitividad, Instituto de Salud Carlos III y Fondos FEDER (Expte. Nº PI12/00498).
MINISTERIO DE ECONOMÍA Y COMPETITIVIDAD
Instituto de Salud Carlos III
Unión Europea
Fondo Europeo de Desarrollo Regional
"Una manera de hacer Europa"
Andalucía se mueve con Europa

8. ***Cursos o talleres de formación**: Formación en conocimientos específicos para mejorar la atención a la persona dependiente.*

9. ***Grupos de apoyo o ayuda mutua**: Ayuda grupal para apoyo, consejo y acompañamiento en el proceso de cuidar. Grupos de "iguales" para ayudarse unos a otros. Estos grupos permiten establecer contacto entre distintos cuidadores, como medio para conseguir apoyo, consejo, protección y acompañamiento durante el proceso de cambio al que se ven sometidos. Son un espacio, un lugar de encuentro, donde los cuidadores se sienten comprendidos, escuchados y apoyados. Ejemplos: asociaciones de familiares y usuarios, talleres de ayuda mutua, etc.*

10. ***Apoyo psicológico y consejo emocional**: Ayuda profesional para desarrollar habilidades y estrategias de afrontamiento de la situación de cuidados. Un/a profesional o terapeuta ayuda a la persona cuidadora a ser consciente de su situación, a reestructurar los pensamientos que mantienen conductas problemáticas, a desarrollar habilidades y estrategias para afrontar los múltiples problemas con que se encuentran, a organizar el tiempo, a llevar a cabo, en la medida de lo posible, actividades gratificantes…*

11. ***Servicio de enfermería a domicilio**: servicio de profesionales de enfermería que acuden a la casa para prestar algún servicio sanitario a la persona cuidadora o la dependiente*

12. ***Servicios de enfermeras/es gestoras/es de casos (también conocidas como enfermeras de enlace)**: Coordinación de todas aquellas actividades dirigidas a la información y formación de las cuidadoras, prestando apoyo a la persona cuidadora de forma individual y grupal. Constituye un eslabón entre la Atención Especializada y la Atención Primaria, teniendo una participación activa en las Comisiones de Cuidados de Área, discusión de casos, sesiones de seguimiento de planes de cuidados conjunto y planificaciones al alta conjunta.*

13. ***Servicios de trabajadores/as o asistentes sociales**: servicios de trabajadores/as o asistentes sociales, como por ejemplo, ayuda para resolver asuntos sobre la casa, pensión o banco.*

14. ***Servicio de comida:** servicio de comidas a domicilio para personas mayores o dependientes.*

15. ***Servicio de transporte**: servicios especiales de transporte a domicilio para acudir a un servicio médico, a un hospital de día, actividades recreativas,….*

María del Mar García Calvente, María del Río Lozano, Isabel Larrañaga Padilla. Cuestionario del Proyecto CUIDAR-SE: Estudio de seguimiento de la salud y la calidad de vida de mujeres y hombres cuidadores en Andalucía y País Vasco. Granada: Escuela Andaluza de Salud Pública; 2019.
Financiado por el Ministerio de Economía y Competitividad, Instituto de Salud Carlos III y Fondos FEDER (Expte. Nº PI12/00498).

MINISTERIO DE ECONOMÍA Y COMPETITIVIDAD
Instituto de Salud Carlos III
Unión Europea
Fondo Europeo de Desarrollo Regional
"Una manera de hacer Europa"
Andalucía se mueve con Europa

J.7. Queremos saber ahora de qué forma necesitaría usted que los servicios sociales y/o sanitarios le apoyaran para cuidar a X. Vea esta relación de propuestas y dígame, por favor, las tres que usted considere más necesarias en primero, segundo y tercer lugar.

	1º	2º	3º
Información y consejo sobre los problemas de las personas a las que cuida y la forma de ayudarle	1	1	1
Información sobre recursos o ayudas disponibles	2	2	2
Apoyo a usted mismo/a para ayudarle a afrontar la situación	3	3	3
Ayudas económicas para facilitarle el cuidado	4	4	4
Alguien que le prestara algunos cuidados concretos a X (ejemplo: aseo, alimentación,…) o que le acompañe en el hogar	5	5	5
Servicios de respiro para disponer de tiempo para otras cosas	6	6	6
Otro tipo de ayudas. Especifique cuál…………………	7	7	7
No necesita ninguna ayuda de los servicios sanitarios y/o sociales para cuidar	8	8	8

J.8. En relación a los servicios sanitarios y/o sociales de los que a usted le gustaría disponer, dígame, por favor, si está usted muy de acuerdo, bastante, poco o nada de acuerdo con las siguientes afirmaciones.

	Muy de acuerdo	Bastante de acuerdo	Poco de acuerdo	Nada de acuerdo	NS/NC
1. Le gustaría disponer de algún centro donde cuidaran a su familiar durante algunas horas al día	1	2	3	4	9
2. Le gustaría disponer de algún centro donde cuidaran de su familiar durante algunos días	1	2	3	4	9
3. Le gustaría disponer de un hogar, residencia o institución similar donde poder llevar a la/s persona/s a las que cuida de forma temporal	1	2	3	4	9
4. Le gustaría disponer de un hogar, residencia o institución similar donde poder llevar a la/s persona/s a las que cuida de forma permanente	1	2	3	4	9

María del Mar García Calvente, María del Río Lozano, Isabel Larrañaga Padilla. Cuestionario del Proyecto CUIDAR-SE: Estudio de seguimiento de la salud y la calidad de vida de mujeres y hombres cuidadores en Andalucía y País Vasco. Granada: Escuela Andaluza de Salud Pública; 2019.
Financiado por el Ministerio de Economía y Competitividad, Instituto de Salud Carlos III y Fondos FEDER (Expte. Nº PI12/00498).

MINISTERIO DE ECONOMÍA Y COMPETITIVIDAD
Instituto de Salud Carlos III
Unión Europea
Fondo Europeo de Desarrollo Regional
"Una manera de hacer Europa"
Andalucía se mueve con Europa

SECCIÓN K. NECESIDADES Y DEMANDAS GLOBALES DE APOYO PARA CUIDAR

K.1. ¿Piensa que, en caso de necesitarlo, podría contar con los siguientes apoyos, ya sean formales o informales, es decir, tanto de los servicios sociales y/o sanitarios como de familiares y otras personas de su entorno social?

	Sí	No	NS/NC
Alguien que cuide a su familiar durante un periodo breve de tiempo (horas, días)	1	2	9
Alguien que cuide a su familiar durante un periodo largo de tiempo (más de una semana)	1	2	9
Alguien que pudiera hacerse cargo de su familiar indefinidamente	1	2	9

K.2. Por favor indique si se encuentra con alguna de las siguientes dificultades en alguna de las tareas que realiza para cuidar a X. Marque con una x todas las opciones que mencione el/la informante.

Percibe especial dificultad por faltarle fuerza física	
Tiene dudas sobre cómo sería la mejor manera de hacerlo	
Piensa que la persona a la que cuida no colabora o se resiste cuando le ayuda con esa labor	
Cree que para llevar a cabo los cuidados que usted realiza necesitaría más formación especializada de la que tiene	
Tiene otras dificultades. Indique cuáles ______________________________	

María del Mar García Calvente, María del Río Lozano, Isabel Larrañaga Padilla. Cuestionario del Proyecto CUIDAR-SE: Estudio de seguimiento de la salud y la calidad de vida de mujeres y hombres cuidadores en Andalucía y País Vasco. Granada: Escuela Andaluza de Salud Pública; 2019.
Financiado por el Ministerio de Economía y Competitividad, Instituto de Salud Carlos III y Fondos FEDER (Expte. Nº PI12/00498).

BLOQUE IV. ESTADO DE SALUD Y CALIDAD DE VIDA DE LA PERSONA CUIDADORA

SECCIÓN L. ESTADO DE SALUD GENERAL

L.1. ¿Recuerda cuál era su estado de salud previo al cuidado (los días o semanas previas a que asumiera el cuidado de X)?

1. Excelente
2. Muy buena
3. Buena
4. Regular
5. Mala
9. NS/NC

L.2. En general, usted diría que su salud es...

1. Excelente
2. Muy buena
3. Buena
4. Regular
5. Mala
9. NS/NC

L.3. Durante las 4 últimas semanas, ¿hasta qué punto el dolor le ha dificultado su trabajo habitual (incluido el de las tareas domésticas)?

1. Nada
2. Un poco
3. Regular
4. Bastante
5. Mucho

L.4. Durante las 4 últimas semanas, ¿con qué frecuencia la salud física o los problemas emocionales le han dificultado sus actividades sociales?

1. Siempre
2. Casi siempre
3. Algunas veces
4. Sólo alguna vez
5. Nunca

Financiado por el Ministerio de Economía y Competitividad, Instituto de Salud Carlos III y Fondos FEDER (Expte. Nº PI12/00498).
MINISTERIO DE ECONOMÍA Y COMPETITIVIDAD
Instituto de Salud Carlos III
Unión Europea
Fondo Europeo de Desarrollo Regional
"Una manera de hacer Europa"
Andalucía se mueve con Europa

SECCIÓN M. MORBILIDAD, SALUD MENTAL Y CONSUMO DE MEDICAMENTOS

M.1. Durante las cuatro últimas semanas, ¿ha padecido usted alguna de las siguientes molestias o dolores?

	Sí	No	NS/NC
Dolor de espalda	1	2	9
Dolor de cabeza o jaqueca	1	2	9
Mareo o vértigo	1	2	9
Dolor en el pecho, ahogo o dificultad para respirar	1	2	9
Dolor de piernas o articulaciones	1	2	9
Dolor de estómago o gastritis	1	2	9
Dolor de regla	1	2	9
Cansancio sin razón aparente	1	2	9

M.2. ¿Tiene alguna enfermedad o problema de salud crónicos o de larga duración? (Entendemos por larga duración si el problema de salud o enfermedad ha durado o se espera que dure 6 meses o más).

1. Sí
2. No
9. NS/NC

M.3. A continuación le voy a leer una lista con una serie de enfermedades o problemas de salud. ¿Padece o ha padecido alguna vez alguna de ellas?

M.3.a. ¿Alguna vez ha padecido…?
Entrevistador/a: léale al informante las enfermedades que se relacionan una a una anotando la respuesta que proceda en la siguiente tabla.

Entrevistador/a: Para las que ha contestado "sí", realizar la M.3.b y anotar en la tabla

M.3.b. ¿La ha padecido en los últimos doce meses?

1. Sí
2. No
9. NS/NC

María del Mar García Calvente, María del Río Lozano, Isabel Larrañaga Padilla. Cuestionario del Proyecto CUIDAR-SE: Estudio de seguimiento de la salud y la calidad de vida de mujeres y hombres cuidadores en Andalucía y País Vasco. Granada: Escuela Andaluza de Salud Pública; 2019.
Financiado por el Ministerio de Economía y Competitividad, Instituto de Salud Carlos III y Fondos FEDER (Expte. Nº PI12/00498).

	¿Alguna vez ha padecido…?			¿La ha padecido en los últimos 12 meses?		
	Sí	No	NS/NC	Sí	No	NS/NC
1. Tensión alta	1	2	9	1	2	9
2. Infarto de miocardio	1	2	9	1	2	9
3. Otras enfermedades del corazón	1	2	9	1	2	9
4. Varices en las piernas	1	2	9	1	2	9
5. Artrosis, artritis o reumatismo	1	2	9	1	2	9
6. Dolor de espalda crónico (cervical)	1	2	9	1	2	9
7. Dolor de espalda crónico (lumbar)	1	2	9	1	2	9
8. Alergia crónica (asma alérgica excluída)	1	2	9	1	2	9
9. Asma	1	2	9	1	2	9
10. Bronquitis crónica, enfisema, enfermedad pulmonar obstructiva crónica (EPOC)	1	2	9	1	2	9
11. Diabetes	1	2	9	1	2	9
12. Úlcera de estómago o duodeno	1	2	9	1	2	9
13. Incontinencia urinaria	1	2	9	1	2	9
14. Colesterol alto	1	2	9	1	2	9
15. Cataratas	1	2	9	1	2	9
16. Problemas crónicos de piel	1	2	9	1	2	9
17. Estreñimiento crónico	1	2	9	1	2	9
18. Cirrosis, disfunción hepática	1	2	9	1	2	9
19. Depresión crónica	1	2	9	1	2	9
20. Ansiedad crónica	1	2	9	1	2	9
21. Otros problemas mentales	1	2	9	1	2	9
22. Embolia, infarto cerebral, hemorragia cerebral	1	2	9	1	2	9
23. Migraña o dolor de cabeza frecuente	1	2	9	1	2	9
24. Hemorroides	1	2	9	1	2	9
25. Tumores malignos	1	2	9	1	2	9
26. Osteoporosis	1	2	9	1	2	9
27. Anemia	1	2	9	1	2	9
28. Problemas de tiroides	1	2	9	1	2	9
29. Problemas de próstata (sólo hombres)	1	2	9	1	2	9
30. Problemas de periodo menopáusico (solo mujeres)	1	2	9	1	2	9
31. Lesiones o defectos permanentes causados por un accidente	1	2	9	1	2	9
32. Ha padecido alguna otra enfermedad crónica …	1	2	9	1	2	9
32.1. …¿Cuál? (1)				1	2	9
32.2. …¿Cuál? (2)				1	2	9
32.3. …¿Cuál? (3)				1	2	9

María del Mar García Calvente, María del Río Lozano, Isabel Larrañaga Padilla. Cuestionario del Proyecto CUIDAR-SE: Estudio de seguimiento de la salud y la calidad de vida de mujeres y hombres cuidadores en Andalucía y País Vasco. Granada: Escuela Andaluza de Salud Pública; 2019.
Financiado por el Ministerio de Economía y Competitividad, Instituto de Salud Carlos III y Fondos FEDER (Expte. Nº PI12/00498).

MINISTERIO DE ECONOMÍA Y COMPETITIVIDAD
Instituto de Salud Carlos III
Unión Europea
Fondo Europeo de Desarrollo Regional
"Una manera de hacer Europa"
Andalucía se mueve con Europa

M.4. Ahora nos gustaría saber cómo se ha sentido, en general, durante las últimas semanas. Por favor, conteste a todas las preguntas indicando la respuesta que, a su juicio, mejor puede aplicarse a usted. Recuerde que sólo debe responder sobre los problemas recientes y los que tiene ahora, no sobre los que tuvo en el pasado. Es importante que intente contestar todas las preguntas.

CUESTIONARIO DE SALUD GENERAL DE GOLDBERG (GHQ-12)[3]

Entrevistador/a, léale al informante: "Voy a entregarle una tarjeta con las preguntas sobre la frecuencia con la que se ha sentido o encontrado como dice cada pregunta. Cuando le pregunte, dígame sólo el número de su respuesta. Tenga en cuenta que las respuestas no son iguales para todas las preguntas". (Entregue la tarjeta y lea una a una cada pregunta y registre el número de la respuesta dada por el entrevistado)
Entrevistador/a, el concepto "últimas semanas" lo debe interpretar el informante como desee.

1. ¿Ha podido concentrarse bien en lo que hacía?
   0. Más que lo habitual
   1. Igual que lo habitual
   2. Menos que lo habitual
   3. Mucho menos que lo habitual
   8. No sabe
   9. No contesta

2. ¿Sus preocupaciones le han hecho perder mucho sueño?
   0. No, en absoluto
   1. No más que lo habitual
   2. Algo más que lo habitual
   3. Mucho más que lo habitual
   8. No sabe
   9. No contesta

3. ¿Ha sentido que está desempeñando un papel útil en la vida?
   0. Más útil que lo habitual
   1. Igual que lo habitual
   2. Menos útil que lo habitual
   3. Mucho menos útil que lo habitual
   8. No sabe
   9. No contesta

4. ¿Se ha sentido capaz de tomar decisiones?
   0. Más que lo habitual

---

[3] Goldberg D. The detection of psychiatric illness by questionnaire. London: Oxford University Press, 1972.

Financiado por el Ministerio de Economía y Competitividad, Instituto de Salud Carlos III y Fondos FEDER (Expte. Nº PI12/00498).
MINISTERIO DE ECONOMÍA Y COMPETITIVIDAD
Instituto de Salud Carlos III
Unión Europea
Fondo Europeo de Desarrollo Regional
"Una manera de hacer Europa"
Andalucía se mueve con Europa

1. Igual que lo habitual
2. Menos que lo habitual
3. Mucho menos que lo habitual
8. No sabe
9. No contesta

5. ¿Se ha notado constantemente agobiado/a y en tensión?
   0. No, en absoluto
   1. No más que lo habitual
   2. Algo más que lo habitual
   3. Mucho más que lo habitual
   8. No sabe
   9. No contesta

6. ¿Ha tenido la sensación de que no puede superar sus dificultades?
   0. No, en absoluto
   1. No más que lo habitual
   2. Algo más que lo habitual
   3. Mucho más que lo habitual
   8. No sabe
   9. No contesta

7. ¿Ha sido capaz de disfrutar de sus actividades normales de cada día?
   0. Más que lo habitual
   1. Igual que lo habitual
   2. Menos que lo habitual
   3. Mucho menos que lo habitual
   8. No sabe
   9. No contesta

8. ¿Ha sido capaz de hacer frente adecuadamente a sus problemas?
   0. Más capaz que lo habitual
   1. Igual que lo habitual
   2. Menos capaz que lo habitual
   3. Mucho menos capaz que lo habitual
   8. No sabe
   9. No contesta

9. ¿Se ha sentido poco feliz o deprimido/a?
   0. No, en absoluto
   1. No más que lo habitual
   2. Algo más que lo habitual
   3. Mucho más que lo habitual
   8. No sabe
   9. No contesta

Financiado por el Ministerio de Economía y Competitividad, Instituto de Salud Carlos III y Fondos FEDER (Expte. Nº PI12/00498).
MINISTERIO DE ECONOMÍA Y COMPETITIVIDAD
Instituto de Salud Carlos III
Unión Europea
Fondo Europeo de Desarrollo Regional
"Una manera de hacer Europa"
Andalucía se mueve con Europa

10. ¿Ha perdido confianza en sí mismo/a?
    0. No, en absoluto
    1. No más que lo habitual
    2. Algo más que lo habitual
    3. Mucho más que lo habitual
    8. No sabe
    9. No contesta

11. ¿Ha pensado que usted es una persona que no vale para nada?
    0. No, en absoluto
    1. No más que lo habitual
    2. Algo más que lo habitual
    3. Mucho más que lo habitual
    8. No sabe
    9. No contesta

12. ¿Se siente razonablemente feliz considerando todas las circunstancias?
    0. Más que lo habitual
    1. Igual que lo habitual
    2. Menos que lo habitual
    3. Mucho menos que lo habitual
    8. No sabe
    9. No contesta

M.5. Entrevistador/a, léale al informante: “Ahora le voy a preguntar sobre los medicamentos que ha consumido en las dos últimas semanas”.
(Hombre) Durante las últimas dos semanas, ¿ha consumido algún medicamento, independientemente de que se lo recetara el médico o no? Por favor, tenga en cuenta también suplementos dietéticos y vitaminas.
(Mujer) Durante las últimas dos semanas, ¿ha consumido algún medicamento, independientemente de que se lo recetara el médico o no? Por favor, tenga en cuenta también suplementos dietéticos, vitaminas, pastillas anticonceptivas y otros medicamentos hormonales.
    1. Sí
    2. No (Pasar a N.7.)

M.6. A continuación voy a leerle una lista de tipos de medicamentos, por favor dígame ¿cuál o cuáles de ellos ha consumido en las últimas dos semanas y cuáles le fueron recetados por el médico/a?
Entrevistador/a, léale al informante la lista de medicamentos y pregúntele, para cada uno de ellos, si los ha consumido o no en las últimas dos semanas y, en caso afirmativo, si le fueron o no recetados por algún médico/a. Para cada medicamento que haya consumido debe cumplimentar la columna Recetado.

María del Mar García Calvente, María del Río Lozano, Isabel Larrañaga Padilla. Cuestionario del Proyecto CUIDAR-SE: Estudio de seguimiento de la salud y la calidad de vida de mujeres y hombres cuidadores en Andalucía y País Vasco. Granada: Escuela Andaluza de Salud Pública; 2019.
Financiado por el Ministerio de Economía y Competitividad, Instituto de Salud Carlos III y Fondos FEDER (Expte. Nº PI12/00498).
MINISTERIO DE ECONOMÍA Y COMPETITIVIDAD
Instituto de Salud Carlos III
Unión Europea
Fondo Europeo de Desarrollo Regional
“Una manera de hacer Europa”
Andalucía se mueve con Europa

	Consumido			Recetado		
	Sí	No	NS/NC	Sí	No	NS/NC
Medicinas para el catarro, gripe, garganta, bronquios	1	2	9	1	2	9
Medicinas para el dolor	1	2	9	1	2	9
Medicinas para bajar la fiebre	1	2	9	1	2	9
Reconstituyentes como vitaminas, minerales, tónicos	1	2	9	1	2	9
Laxantes	1	2	9	1	2	9
Antibióticos	1	2	9	1	2	9
Tranquilizantes, relajantes, pastillas para dormir	1	2	9	1	2	9
Medicamentos para la alergia	1	2	9	1	2	9
Medicamentos para la diarrea	1	2	9	1	2	9
Medicinas para el reuma	1	2	9	1	2	9
Medicinas para el corazón	1	2	9	1	2	9
Medicinas para la tensión arterial	1	2	9	1	2	9
Medicinas para el estómago y/o las alteraciones digestivas	1	2	9	1	2	9
Antidepresivos, estimulantes	1	2	9	1	2	9
Píldoras para no quedar embarazada (sólo mujeres)	1	2	9	1	2	9
Hormonas para la menopausia (sólo mujeres)	1	2	9	1	2	9
Medicamentos para adelgazar	1	2	9	1	2	9
Medicamentos para bajar colesterol	1	2	9	1	2	9
Medicamentos para la diabetes	1	2	9	1	2	9
Medicamentos para el tiroides	1	2	9	1	2	9
Otros medicamentos	1	2	9	1	2	9

M.7. Dígame también si ha consumido en las últimas dos semanas…

Productos homeopáticos: Si (1) No (2) NS/NC (9)
Productos naturistas: Si (1) No (2) NS/NC (9)

SECCIÓN N. UTILIZACIÓN DE SERVICIOS SANITARIOS

A continuación voy a hacerle algunas preguntas sobre la utilización de los servicios sanitarios para usted mismo/a

N.1. En las últimas cuatro semanas, ¿ha consultado con un médico/a de familia o médico/a general por algún problema, molestia o enfermedad suya?

1. Sí ----------------Ñ.2. ¿Cuántas veces?
2. No
9. NS/NC

María del Mar García Calvente, María del Río Lozano, Isabel Larrañaga Padilla. Cuestionario del Proyecto CUIDAR-SE: Estudio de seguimiento de la salud y la calidad de vida de mujeres y hombres cuidadores en Andalucía y País Vasco. Granada: Escuela Andaluza de Salud Pública; 2019.
Financiado por el Ministerio de Economía y Competitividad, Instituto de Salud Carlos III y Fondos FEDER (Expte. Nº PI12/00498).
MINISTERIO DE ECONOMÍA Y COMPETITIVIDAD
Instituto de Salud Carlos III
Unión Europea
Fondo Europeo de Desarrollo Regional
"Una manera de hacer Europa"
Andalucía se mueve con Europa

N.3. ¿Y con un/a especialista?, ¿ha consultado en las últimas cuatro semanas por algún problema, molestia o enfermedad suya?

1. Sí ----------------N.4. ¿Cuántas veces?
2. No
9. NS/NC

Sólo si ha contestado "sí" en N.1 o N.3: realizar N.5, N.6 y N.7

N.5.

¿Cuál fue el motivo principal de la última consulta?

1. Diagnóstico de una enfermedad o problema de salud
2. Un accidente o agresión
3. Revisión
4. Sólo dispensación de recetas
5. Parte de baja, confirmación o alta
6. Otros motivos
9. NS/NC

N.6. Y esta última vez, ¿cuánto tiempo pasó desde que empezó a notarse enfermo/a o sintió que tenía algún problema de salud hasta que pidió cita para consulta?
(Entrevistador/a: Si es menos de un mes, conteste en días. Si es menos de un día, conteste en horas).

_ _ Meses
_ _ Días
_ _ Horas
9. NS/NC

N.7. Y también esta última vez, ¿cuánto tiempo pasó desde que pidió la cita hasta que le vio el/la médico/a?
(Entrevistador/a: Si es menos de un mes, conteste en días. Si es menos de un día, conteste en horas).

_ _ Meses
_ _ Días
_ _ Horas
9. NS/NC

N-8. ¿Cuánto tiempo hace que acudió al dentista, estomatólogo o higienista dental para examen, consejo o tratamiento de problemas de su dentadura o boca?

1. Hace 3 meses o menos
2. Hace más de 3 meses y menos de 12 meses (pasar a N.10)
3. Hace un año o más (pasar a N.10)
4. Nunca ha ido (pasar a N.10)
9. NS/NC

N.9. ¿Cuántas veces ha acudido en los últimos 3 meses? _ _ 99 _ (NS/NC)

Financiado por el Ministerio de Economía y Competitividad, Instituto de Salud Carlos III y Fondos FEDER (Expte. Nº PI12/00498).
MINISTERIO DE ECONOMÍA Y COMPETITIVIDAD
Instituto de Salud Carlos III
Unión Europea
Fondo Europeo de Desarrollo Regional
"Una manera de hacer Europa"
Andalucía se mueve con Europa

N.10. Durante los últimos 12 meses, ¿ha tenido que ingresar en un hospital como paciente durante al menos una noche?

1. Sí ----------------N.11. ¿Cuántas veces? _ _ 99 _ (NS/NC)
2. No (Pasar a N.13)
9. NS/NC (Pasar a N.13)

N.12. En relación a su último ingreso hospitalario ocurrido en los últimos 12 meses ¿Cuántos días estuvo ingresado/a en el hospital?

Nº días: _ _ 99 _ (NS/NC)

N.13. Durante los últimos doce meses, desde el (fecha de hace un año) ¿ha sido usted atendido/a en un "Hospital de día" para una intervención, tratamiento o hacerse alguna prueba, es decir, permaneciendo durante todo o parte del día pero sin tener que pasar allí la noche? (Incluya ingresos en cama o sillón-cama, no incluya estancias en urgencias ni en observación)

1. Sí ----------------N.14. ¿Cuántas veces? _ _ 99 _ (NS/NC)
2. No (Pasar a N.16)
9. NS/NC (Pasar a N.16)

N.15. ¿Cuántos días ha tenido que acudir a un hospital de día, sin tener que pasar la noche, desde el (fecha de hace un año)?

Nº días: _ _ 99 _ (NS/NC)

N.16. En estos últimos 12 meses, ¿ha tenido que utilizar algún servicio de urgencias por algún problema o enfermedad?

1. Sí ----------------N.17. ¿Cuántas veces? _ _ 99 _ (NS/NC)
2. No (Pasar a N.19)
9. NS/NC (Pasar a N.19)

N.18. La última vez que utilizó un servicio de urgencias, ¿cuánto tiempo pasó desde que empezó a notarse enfermo/a o sintió que tenía algún problema de salud hasta que pidió asistencia?
(Entrevistador/a: Si es menos de un día, conteste en horas. Si es menos de una hora, conteste en minutos).

_ _ Días
_ _ Horas
_ _ Minutos
9. NS/NC

N.19. En los últimos 12 meses, ¿alguna vez ha necesitado asistencia médica y no la ha recibido?

1. Sí
2. No (Pasar a sección O)

María del Mar García Calvente, María del Río Lozano, Isabel Larrañaga Padilla. Cuestionario del Proyecto CUIDAR-SE: Estudio de seguimiento de la salud y la calidad de vida de mujeres y hombres cuidadores en Andalucía y País Vasco. Granada: Escuela Andaluza de Salud Pública; 2019.
Financiado por el Ministerio de Economía y Competitividad, Instituto de Salud Carlos III y Fondos FEDER (Expte. Nº PI12/00498).
MINISTERIO DE ECONOMÍA Y COMPETITIVIDAD
Instituto de Salud Carlos III
Unión Europea
Fondo Europeo de Desarrollo Regional
"Una manera de hacer Europa"
Andalucía se mueve con Europa

9. NS/NC (Pasar a sección O)

N.20. ¿Cuál ha sido la causa principal por la que no ha recibido esa asistencia? Entrevistador/a, no se leen las opciones. Señale aquélla que le facilite espontáneamente, siempre que coincida con alguna de las incluidas en las opciones 1 a 6. Si la respuesta no coincide con ninguna de estas opciones, señale la alternativa 7 "Otras razones".

1. No me lo podía permitir (demasiado caro o no cubierto por el seguro)
2. Había que esperar demasiado
3. No disponía de tiempo debido al trabajo
4. No disponía de tiempo debido al cuidado de menores o de otras personas
4. Demasiado lejos para viajar/sin medios de transporte
5. Miedo al médico/hospitales/exploraciones médicas/tratamiento
6. Quise esperar y ver si el problema mejoraba por sí solo
7. Otras razones
8. NS
9. NC

SECCIÓN O. PRÁCTICAS PREVENTIVAS

O.1. ¿Se ha vacunado de la gripe en la última campaña?

1. Sí
2. No
9. NS/NC

O.2. Le ha tomado la tensión alguna vez un/a profesional sanitario/a, excluyendo las tomas de tensión en farmacias?

1. Sí
2. No (pasar a O.4)
9. NC (pasar a O.4)

O.3. La última vez que le tomaron la tensión fue:

1. En los últimos doce meses
2. Hace más de un año pero no más de 2 años
3. Hace más de 2 años pero no más de 5 años
4. Hace más de 5 años
5. Nunca se la ha tomado
9. NS/NC

O.4. ¿Le han medido alguna vez su nivel de colesterol por prescripción médica, excluyendo las mediciones en farmacias?

1. Sí
2. No (Mujeres pasan a O.6; hombres pasan a la siguiente sección)
9. NS/NC (Mujeres pasan a O.6; hombres pasan a la siguiente sección)

María del Mar García Calvente, María del Río Lozano, Isabel Larrañaga Padilla. Cuestionario del Proyecto CUIDAR-SE: Estudio de seguimiento de la salud y la calidad de vida de mujeres y hombres cuidadores en Andalucía y País Vasco. Granada: Escuela Andaluza de Salud Pública; 2019.
Financiado por el Ministerio de Economía y Competitividad, Instituto de Salud Carlos III y Fondos FEDER (Expte. Nº PI12/00498).
MINISTERIO DE ECONOMÍA Y COMPETITIVIDAD
Instituto de Salud Carlos III
Unión Europea
Fondo Europeo de Desarrollo Regional
"Una manera de hacer Europa"
Andalucía se mueve con Europa

O. 5. La última vez que se lo midieron:

1. En los últimos doce meses
2. Hace más de un año pero no más de 5 años
3. Hace más de 5 años
9. NS/NC

[Siguientes de esta sección: Sólo mujeres]

O.6. ¿Cuándo ha efectuado la última visita a una consulta ginecológica por algún motivo distinto a los relacionados con embarazo o parto?

1. Hace menos de 6 meses
2. Entre 6 meses y 1 año
3. Entre 1 y 3 años
4. Hace más de 3 años
5. Nunca ha ido por motivos diferentes a embarazo o parto
9. NS/NC

O.7. ¿Le han hecho alguna vez una mamografía?

1. Sí
2. No (pasar a O.9)
9. NC (pasar a O.9)

O.8. ¿Cuándo fue la última vez que le hicieron una mamografía (radiografía de mama)?

1. En los últimos doce meses
2. Hace más de un año y menos de 2
3. Hace más de 2 años y menos de 3
4. Hace más de 3 años
9. NS/NC

O.9. ¿Le han hecho alguna vez una citología vaginal (muestra de células)?

1. Sí
2. No (pasar a siguiente sección)
9. NC (pasar a siguiente sección)

O.8. ¿Cuándo fue la última vez que le hicieron una citología vaginal?

1. Hace 3 años o menos
2. Hace más de 3 años pero no más de 5 años
3. Hace más de 5 años
9. NS/NC

María del Mar García Calvente, María del Río Lozano, Isabel Larrañaga Padilla. Cuestionario del Proyecto CUIDAR-SE: Estudio de seguimiento de la salud y la calidad de vida de mujeres y hombres cuidadores en Andalucía y País Vasco. Granada: Escuela Andaluza de Salud Pública; 2019.
Financiado por el Ministerio de Economía y Competitividad, Instituto de Salud Carlos III y Fondos FEDER (Expte. Nº PI12/00498).
MINISTERIO DE ECONOMÍA Y COMPETITIVIDAD
Instituto de Salud Carlos III
Unión Europea
Fondo Europeo de Desarrollo Regional
"Una manera de hacer Europa"
Andalucía se mueve con Europa

SECCIÓN P. HÁBITOS SALUDABLES

P.1. ¿Podría decirme cuánto pesa, aproximadamente, sin zapatos ni ropa?
Peso en kg | _ _ _ | NS/NC 999

P.2. ¿Y cuánto mide, aproximadamente, sin zapatos?
Altura en cm | _ _ _ | NS/NC 999

P.3. Y, en relación a su estatura, diría que su peso es:
1. Bastante mayor de lo normal
2. Algo mayor de lo normal
3. Normal
4. Menor de lo normal
9. NS/NC

P.4. Le preguntaré sobre el consumo de tabaco. ¿Podría decirme si actualmente fuma?
1. Sí, fuma diariamente
2. Sí fuma, pero no diariamente (pasar a P.6)
3. No fuma actualmente, pero ha fumado antes (pasar a P.6)
4. No fuma, ni ha fumado nunca de manera habitual (pasar a P.6)
9. NS/NC (pasar a P.6)

P.5. ¿Diría que fuma ahora más, menos o igual que hace un año? Entrevistador/a: no se leen las opciones
1. Más
2. Menos
3. Igual
9. NS/NC

P.6. Ahora quisiera hacerle unas preguntas respecto al consumo de bebidas alcohólicas, es decir, cualquier tipo de bebida que contenga alcohol, independientemente de su graduación. ¿Ha tomado Vd. en estas últimas dos semanas alguna bebida alcohólica?
1. Sí (Pasar a P.8)
2. No
9. NS/NC

P.7. ¿Ha tomado Vd. en los últimos 12 meses alguna bebida alcohólica?
1. Sí
2. No
9. NS/NC

María del Mar García Calvente, María del Río Lozano, Isabel Larrañaga Padilla. Cuestionario del Proyecto CUIDAR-SE: Estudio de seguimiento de la salud y la calidad de vida de mujeres y hombres cuidadores en Andalucía y País Vasco. Granada: Escuela Andaluza de Salud Pública; 2019.
Financiado por el Ministerio de Economía y Competitividad, Instituto de Salud Carlos III y Fondos FEDER (Expte. Nº PI12/00498).

P.8. ¿Podría indicarme, aproximadamente, cuántas horas duerme habitualmente al día? Incluya las horas de siesta.

Nº de horas diarias | _ _ | NS/NC ☐ 99

P.9. ¿Descansa cuando duerme?

1. Sí, descansa bien
2. No descansa lo suficiente
9. NS/NC

P.10. ¿Tiene dificultades para dormir? (conciliar el sueño)

1. Sí
2. No
9. NS/NC

P.11. ¿Cuál de estas posibilidades describe mejor la frecuencia con la que realiza alguna actividad física en su tiempo libre?

1. No hago ejercicio. El tiempo libre lo ocupo de forma casi completamente sedentaria (leer, ver la televisión, ir al cine, etc.)
2. Hago alguna actividad física o deportiva ocasional (caminar o pasear, bicicleta, jardinería, gimnasia suave, actividades recreativas que requieren un ligero esfuerzo, etc.)
3. Hago actividad física varias veces al mes (deportes, gimnasia, correr, natación, ciclismo, juegos de equipo, etc.)
4. Hago entrenamiento deportivo o físico varias veces a la semana
9. NS/NC

P.12. ¿Con qué frecuencia consume los siguientes tipos de alimentos?

Tipos de alimentos	Frecuencias de consumo					
	A diario	3 o más veces a la semana, pero no a diario	1 o 2 veces a la semana	Menos de 1 vez a la semana	Nunca o casi nunca	NS/NC
Verduras, ensaladas, hortalizas y fruta fresca	1	2	3	4	5	9
Carne	1	2	3	4	5	9
Huevos	1	2	3	4	5	9
Pescado	1	2	3	4	5	9
Productos lácteos	1	2	3	4	5	9

Financiado por el Ministerio de Economía y Competitividad, Instituto de Salud Carlos III y Fondos FEDER (Expte. Nº PI12/00498).
MINISTERIO DE ECONOMÍA Y COMPETITIVIDAD
Instituto de Salud Carlos III
Unión Europea
Fondo Europeo de Desarrollo Regional
"Una manera de hacer Europa"
Andalucía se mueve con Europa

SECCIÓN Q. SATISFACCIÓN CON LA VIDA

Q.1. ¿Podría decirme si en la actualidad está muy satisfecho/a, bastante, poco o nada satisfecho/a con los siguientes aspectos de su vida?

	Muy satisfecho/a	Bastante satisfecho/a	Poco satisfecho/a	Nada satisfecho/a
1. La situación de su casa y su familia	1	2	3	4
2. Sus condiciones laborales	1	2	3	4
3. Condiciones de su vivienda	1	2	3	4
4. Su situación económica	1	2	3	4
5. Su salud y bienestar	1	2	3	4
6. Su tiempo libre	1	2	3	4
7. Su círculo de amigos y conocidos	1	2	3	4
8. El apoyo que recibe de los servicios sanitarios, sociales o de voluntariado	1	2	3	4

Q.2. Teniendo en cuenta todo lo que le ocurre en este momento, en general, ¿hasta qué punto se siente usted satisfecho/a con la vida que lleva?

1. Muy satisfecho/a
2. Bastante satisfecho/a
3. Poco satisfecho/a
4. Nada satisfecho/a
9. NC

CALIDAD DE VIDA RELACIONADA CON LA SALUD (ESCALA EQ-5D-5L)[4]

Introducción: Entrevistador/a, léale al informante: “Continuando con su estado de salud actual, dígame qué afirmaciones de las siguientes que le voy a leer describe mejor su estado de salud en el día de hoy”:

(No leer: Movilidad)

No tengo problemas para caminar (1)

[4] Las variables de calidad de vida relacionada con la salud se obtienen con un instrumento de medida propiedad del Grupo EuroQol: © 2009 EuroQol Group. EQ-5D-5L™ is a trademark of the EuroQol Group. EuroQol Group (1990). EuroQol—a new facility for the measurement of health-related quality of life. Health Policy, 16(3), 199–208.

Financiado por el Ministerio de Economía y Competitividad, Instituto de Salud Carlos III y Fondos FEDER (Expte. Nº PI12/00498).
MINISTERIO DE ECONOMÍA Y COMPETITIVIDAD
Instituto de Salud Carlos III
Unión Europea
Fondo Europeo de Desarrollo Regional
“Una manera de hacer Europa”
Andalucía se mueve con Europa

Tengo problemas leves para caminar (2)
Tengo problemas moderados para caminar (3)
Tengo problemas graves para caminar (4)
No puedo caminar (5)
No sabe (8)
No contesta (9)

(No leer: Autocuidado)

No tengo problemas para lavarme o vestirme (1)
Tengo problemas leves para lavarme o vestirme (2)
Tengo problemas moderados para lavarme o vestirme (3)
Tengo problemas graves para lavarme o vestirme (4)
No puedo lavarme o vestirme (5)
No sabe (8)
No contesta (9)

(No leer: Actividades cotidianas, por ejemplo, trabajar, estudiar, hacer las tareas domésticas, actividades familiares o actividades durante el tiempo libre)

No tengo problemas para realizar mis actividades cotidianas (1)
Tengo problemas leves para realizar mis actividades cotidianas (2)
Tengo problemas moderados para realizar mis actividades cotidianas (3)
Tengo problemas graves para realizar mis actividades cotidianas (4)
No puedo realizar mis actividades cotidianas (5)
No sabe (8)
No contesta (9)

(No leer: Dolor/malestar)

No tengo dolor ni malestar (1)
Tengo dolor o malestar leve (2)
Tengo dolor o malestar moderado (3)
Tengo dolor o malestar fuerte (4)
Tengo dolor o malestar extremo (5)
No sabe (8)
No contesta (9)

(No leer: Ansiedad/depresión)

No estoy ansioso/a ni deprimido/a (1)
Estoy levemente ansioso/a o deprimido/a (2)
Estoy moderadamente ansioso/a o deprimido/a (3)
Estoy muy ansioso/a o deprimido/a (4)
Estoy extremadamente ansioso/a o deprimido/a (5)
No sabe (8)
No contesta (9)

María del Mar García Calvente, María del Río Lozano, Isabel Larrañaga Padilla. Cuestionario del Proyecto CUIDAR-SE: Estudio de seguimiento de la salud y la calidad de vida de mujeres y hombres cuidadores en Andalucía y País Vasco. Granada: Escuela Andaluza de Salud Pública; 2019.
Financiado por el Ministerio de Economía y Competitividad, Instituto de Salud Carlos III y Fondos FEDER (Expte. Nº PI12/00498).
MINISTERIO DE ECONOMÍA Y COMPETITIVIDAD
Instituto de Salud Carlos III
Unión Europea
Fondo Europeo de Desarrollo Regional "Una manera de hacer Europa"
Andalucía se mueve con Europa

Nos gustaría conocer lo buena o mala que es su salud HOY.

La siguiente escala está numerada del 0 al 100. Donde:

- 100 representa la mejor salud que usted se pueda imaginar.
- 0 representa la peor salud que usted se pueda imaginar.

Por favor, marque en la escala lo buena o mala que es su salud HOY.

-100 (La mejor salud que usted se pueda imaginar)
-95
-90
-85
-80
-75
-70
-65
-60
-55
-50
-45
-40
-35
-30
-25
-20
-15
-10
-5
-0 (La peor salud que usted se pueda imaginar)

María del Mar García Calvente, María del Río Lozano, Isabel Larrañaga Padilla. Cuestionario del Proyecto CUIDAR-SE: Estudio de seguimiento de la salud y la calidad de vida de mujeres y hombres cuidadores en Andalucía y País Vasco. Granada: Escuela Andaluza de Salud Pública; 2019.
Financiado por el Ministerio de Economía y Competitividad, Instituto de Salud Carlos III y Fondos FEDER (Expte. Nº PI12/00498).

BLOQUE V. CONSECUENCIAS PERCIBIDAS DEL CUIDADO EN LA SALUD Y OTRAS DIMENSIONES DE LA CALIDAD DE VIDA

SECCIÓN R. CONSECUENCIAS GENERALES DEL CUIDADO EN LA SALUD

Entrevistador/a, El hecho de tener que responsabilizarse de una persona que requiere cuidados, puede tener cierta incidencia en el desarrollo de la vida normal de una persona. En estas tarjetas hay una serie de consecuencias que pueden tener este tipo de situaciones. Me gustaría que nos indicara aquéllas que a Vd. Le ocurren por tener que ayudar a X.

R.1. Respecto a aspectos relativos a su salud o estado general, por favor indique si:
Entrevistador/a: Muestre la Tarjeta al/a la entrevistado/a y marque todas las respuestas que mencione:

R.1.a. Se ha deteriorado su salud
R.1.b. Ha tenido que ponerse en tratamiento para poder llevar la situación (tratamiento antidepresivos, de ansiedad/angustia, nervios,....)
R.1.c. Se encuentra cansado/a
R.1.d. Se siente deprimido/a
R.1.e. Tiene otros problemas, pero no los anteriores
R.1.f. No tiene ningún problema
R.1.g. NS/NC

SECCIÓN S: ASPECTOS PROFESIONALES O LABORALES

S.1. Respecto a aspectos profesionales o económicos, por favor indique si…
Entrevistador/a: Muestre la tarjeta al/a informante y marque todas las respuestas que mencione.

No puede plantearse trabajar fuera de casa	
Ha tenido que dejar de trabajar temporalmente	
Ha tenido que dejar de trabajar definitivamente	
Ha tenido que reducir su jornada de trabajo	
Ha tenido que modificar su horario de trabajo	
Tiene problemas para cumplir sus horarios	
Su vida profesional se ha resentido	
No tiene ningún problema	

S.2. ¿En qué medida le resulta fácil compaginar su trabajo remunerado con los cuidados familiares?
1. Muy fácil
2. Bastante fácil

María del Mar García Calvente, María del Río Lozano, Isabel Larrañaga Padilla. Cuestionario del Proyecto CUIDAR-SE: Estudio de seguimiento de la salud y la calidad de vida de mujeres y hombres cuidadores en Andalucía y País Vasco. Granada: Escuela Andaluza de Salud Pública; 2019.
Financiado por el Ministerio de Economía y Competitividad, Instituto de Salud Carlos III y Fondos FEDER (Expte. Nº PI12/00498).
MINISTERIO DE ECONOMÍA Y COMPETITIVIDAD
Instituto de Salud Carlos III
Unión Europea
Fondo Europeo de Desarrollo Regional
"Una manera de hacer Europa"
Andalucía se mueve con Europa

3. Poco fácil
4. Nada fácil
9. NS/NC

S.3. ¿Cuáles de las situaciones que le leo a continuación le permiten compaginar su trabajo con el cuidado de las personas que tiene a su cargo?

	Sí	No	NS/NC
Tiene familia o amigos que le ayudan a cuidar	1	2	9
Cuenta con alguien a quien paga para que le ayude	1	2	9
Tiene un tipo de trabajo que le permite compaginar las dos cosas	1	2	9
Cuenta con otras posibilidades	1	2	9

SECCIÓN T: ASPECTOS ECONÓMICOS

T.1. ¿En qué medida considera que tiene problemas económicos como consecuencia del cuidado?
1. Mucho
2. Bastante
3. Poco
4. Nada
9. NS/NC

T.2. Me gustaría que me dijera si el cuidado de X le ocasiona gastos extras en alguna de las cosas que le cito a continuación.

	Sí	No	NC
En comida o ropa especial	1	2	9
En consulta médica, medicinas o gastos de farmacia	1	2	9
En tratamientos psicológicos, educación especial, logopedia, rehabilitación u otros tratamientos especiales	1	2	9
En transporte (autobús, taxi, etc.)	1	2	9
En acondicionar la vivienda a las necesidades de la persona que cuida	1	2	9
En contratar a alguien para que le acompañe o cuide	1	2	9
Otros (especificar)	1	2	9

T.3. En el último mes, ¿cuánto dinero diría usted que se ha gastado en relación al cuidado de la salud de X?
Euros:

SECCIÓN U. ASPECTOS DE OCIO, TIEMPO LIBRE Y VIDA FAMILIAR

María del Mar García Calvente, María del Río Lozano, Isabel Larrañaga Padilla. Cuestionario del Proyecto CUIDAR-SE: Estudio de seguimiento de la salud y la calidad de vida de mujeres y hombres cuidadores en Andalucía y País Vasco. Granada: Escuela Andaluza de Salud Pública; 2019.
Financiado por el Ministerio de Economía y Competitividad, Instituto de Salud Carlos III y Fondos FEDER (Expte. Nº PI12/00498).
MINISTERIO DE ECONOMÍA Y COMPETITIVIDAD
Instituto de Salud Carlos III
Unión Europea
Fondo Europeo de Desarrollo Regional
"Una manera de hacer Europa"
Andalucía se mueve con Europa

U.1. ¿En qué medida o grado considera que le ocurre lo siguiente, como consecuencia del cuidado?

	Mucho	Bastante	Poco	Nada	NS/NC
Ha tenido que reducir su tiempo de ocio					
No puede ir de vacaciones					
No dispone de tiempo para cuidar de otras personas como le gustaría (por ej. los hijos)					
No tiene tiempo para frecuentar a amistades					
No tiene tiempo para cuidar de sí mismo/a					
No ha podido formar una familia como le hubiera gustado					
No ha tenido los hijos que le hubiera gustado					
Ha empeorado su relación con la persona a la que cuida					
Ha empeorado su relación con su pareja					
Ha empeorado su relación con otros miembros de la familia					

SECCIÓN V. PERCEPCIÓN DE EFECTOS POSITIVOS DEL CUIDADO

Escala de Satisfacción con el cuidado

Me gustaría hablar acerca de algunos sentimientos que pueda tener cuando cuida a esta persona.

V.1. Por favor, dígame en qué medida está de acuerdo con la siguiente afirmación: Siento satisfacción por ayudar a la persona a la que cuido

1. Totalmente de acuerdo
2. Algo de acuerdo
3. Ni de acuerdo no en desacuerdo
4. Algo en desacuerdo
5. Totalmente en desacuerdo

María del Mar García Calvente, María del Río Lozano, Isabel Larrañaga Padilla. Cuestionario del Proyecto CUIDAR-SE: Estudio de seguimiento de la salud y la calidad de vida de mujeres y hombres cuidadores en Andalucía y País Vasco. Granada: Escuela Andaluza de Salud Pública; 2019.
Financiado por el Ministerio de Economía y Competitividad, Instituto de Salud Carlos III y Fondos FEDER (Expte. Nº PI12/00498).

V.2. Dígame, ¿Con qué frecuencia siente lo que le voy a mencionar a continuación?

	Casi siempre	Con bastante frecuencia	Alguna vez	Rara vez	Nunca
1. Ayudar a esta persona le ha hecho sentirse más cercano a ella	5	4	3	2	1
2. Realmente disfruta estando con esta persona	5	4	3	2	1
3. El hecho de responsabilizarse de esta persona le ayuda a aumentar su autoestima	5	4	3	2	1
4. Los momentos placenteros de esta persona le producen placer a usted también	5	4	3	2	1
5. Cuidar a esta persona da un mayor sentido a su vida	5	4	3	2	1

María del Mar García Calvente, María del Río Lozano, Isabel Larrañaga Padilla. Cuestionario del Proyecto CUIDAR-SE: Estudio de seguimiento de la salud y la calidad de vida de mujeres y hombres cuidadores en Andalucía y País Vasco. Granada: Escuela Andaluza de Salud Pública; 2019.
Financiado por el Ministerio de Economía y Competitividad, Instituto de Salud Carlos III y Fondos FEDER (Expte. Nº PI12/00498).
MINISTERIO DE ECONOMÍA Y COMPETITIVIDAD
Instituto de Salud Carlos III
Unión Europea
Fondo Europeo de Desarrollo Regional
"Una manera de hacer Europa"
Andalucía se mueve con Europa

BLOQUE VI. VALORACIÓN CONTINGENTE DEL CUIDADO[5]

SECCIÓN W. VALORACIÓN ECONÓMICA DEL CUIDADO

A continuación, le pedimos que considere todos los aspectos relacionados con los cuidados que Ud. presta a la persona que recibe sus cuidados (estado de salud de esa persona, número de horas/días de cuidados, tareas que presta y duración de las mismas, satisfacciones y consecuencias negativas que para Ud. tiene cuidar de él/ella, etc.).

Suponga que esa persona precisara de su ayuda durante <u>una hora más cada día</u>.

(Entrevistador/a: al 50% de las personas encuestadas debe planteársele primero el Escenario DAP y luego el DAC, y al otro 50% al revés, primero el DAC y luego el DAP). Registrar cuál es el primer escenario que se le plantea).

Primer escenario planteado:
DAP (Disposición a pagar)
DAC (Disposición a ser compensado)

<u>Escenario de Disposición a ser Compensado</u>

W.1. Suponga que el Estado le pide a Ud. que cuide durante esa hora adicional a la persona a la que ya atiende, a cambio de ofrecerle una compensación económica por prestar esa hora extra. ¿Qué cantidad mínima de dinero cree que le haría sentirse compensado/a por tener que prestar esa hora adicional diaria?

Para ayudarle a contestar, le vamos a presentar varias tarjetas con una cantidad de dinero impresa en ellas. Por favor, forme tres grupos con estas tarjetas, según considere que el importe mostrado en ellas:
1. Seguro que sería una compensación suficiente
2. Seguro que sería una compensación insuficiente
3. No sabe si sería una compensación suficiente o no

(Revisar las respuestas para evitar posibles contradicciones. Por ejemplo, no puede señalar que una compensación de 8 € diarios sería suficiente y al mismo tiempo indicar que 10 € al día serían insuficientes o no sabe si serían suficientes o no)

---

[5] Las preguntas relacionadas con la aplicación de valoración contingente han sido adaptadas del cuestionario desarrollado por Garrido-García S, Sánchez-Martínez FI, Abellán-Perpiñán JM, van Exel J. Monetary Valuation of Informal Care Based on Carers' and Noncarers' Preferences. Value Health. 2015 Sep;18(6):832-40.

Financiado por el Ministerio de Economía y Competitividad, Instituto de Salud Carlos III y Fondos FEDER (Expte. Nº PI12/00498).
MINISTERIO DE ECONOMÍA Y COMPETITIVIDAD
Instituto de Salud Carlos III
Unión Europea
Fondo Europeo de Desarrollo Regional
"Una manera de hacer Europa"
Andalucía se mueve con Europa

[Mostrar las tarjetas aleatoriamente, tras haberlas barajado. Anotar las respuestas en la siguiente tabla, marcando con una X en la columna correspondiente]

Importe diario	Importe mensual equivalente	Seguro que sería suficiente	Seguro que no sería suficiente	No sé si sería suficiente o no
0 €	0 €			
1 €	30 €			
2 €	60 €			
3 €	90 €			
4 €	120 €			
5 €	150 €			
6 €	180 €			
8 €	240 €			
10 €	300 €			
12 €	360 €			
15 € (*)	450 € (*)			

[*Si responde que "Seguro que sería suficiente" una compensación de 0 €, pasar a U.2. Si responde que "Seguro sería insuficiente" una compensación de 15 € diarios (450 € mensuales), formular la pregunta U.3. En cualquier otro caso, saltar a las siguientes preguntas*].

W.2. [Sólo a quienes digan que "Seguro sería suficiente" una compensación de 0 euros]

Ud. acaba de decir que está seguro de que 0 euros serían suficientes para compensarle por cuidar a esa persona durante una hora más al día. ¿Podría Ud. decirme por qué motivo no creería necesario recibir compensación económica alguna por esa hora adicional de cuidados?

1. ☐ Cuidar a esa persona durante una hora más al día no me supondría una diferencia tan grande como para necesitar ser compensado/a por ello
2. ☐ Es una cuestión de principios (me sentiría mal aceptando dinero a cambio de cuidar a esa persona)
3. ☐ Otros motivos (especificar): ………………………………….

W.3. [Sólo a quienes respondan que "Seguro sería insuficiente" una compensación de 15 € al día]

Por favor, ¿podría indicar la cantidad mínima diaria (o mensual) que Ud. consideraría suficiente para ser compensado/a por cuidar de esa persona durante una hora más cada día?

……………€/día ó ……….€/mes

María del Mar García Calvente, María del Río Lozano, Isabel Larrañaga Padilla. Cuestionario del Proyecto CUIDAR-SE: Estudio de seguimiento de la salud y la calidad de vida de mujeres y hombres cuidadores en Andalucía y País Vasco. Granada: Escuela Andaluza de Salud Pública; 2019.
Financiado por el Ministerio de Economía y Competitividad, Instituto de Salud Carlos III y Fondos FEDER (Expte. Nº PI12/00498).

MINISTERIO DE ECONOMÍA Y COMPETITIVIDAD
Instituto de Salud Carlos III
Unión Europea
Fondo Europeo de Desarrollo Regional
"Una manera de hacer Europa"
Andalucía se mueve con Europa

[Pueden indicar el importe diario o mensual, según les resulte más sencillo]

Escenario de Disposición a Pagar

W.4. Suponga ahora que el Estado le facilita la ayuda de un cuidador profesional para esa hora diaria extra, pero a cambio usted debe pagar una parte, ¿cuál sería la cantidad máxima de dinero que estaría dispuesto a pagar por ese servicio?

Vamos a mostrarle varias tarjetas muy similares a las anteriores con una cantidad de dinero impresa en ellas. Por favor, forme tres grupos con estas tarjetas, según considere que el importe mostrado en ellas:

1. Pagaría seguro esta cantidad
2. No pagaría seguro más de esta cantidad
3. No sé si pagaría o no esta cantidad

(Revisar las respuestas para evitar posibles contradicciones. Por ejemplo, no puede señalar que no pagaría más de 8 € diarios y al mismo tiempo indicar que pagaría seguro 10 € al día o no sabe si pagaría 10 €)

[Mostrar las tarjetas aleatoriamente, tras haberlas barajado. Anotar las respuestas en la siguiente tabla, marcando con una X en la columna correspondiente]

Importe diario	Importe mensual equivalente	Pagaría seguro esta cantidad	No pagaría seguro más de esta cantidad	No sé si pagaría o no esta cantidad
0 €	0 €			
1 €	30 €			
2 €	60 €			
3 €	90 €			
4 €	120 €			
5 €	150 €			
6 €	180 €			
8 €	240 €			
10 €	300 €			
12 €	360 €			
15 € (*)	450 € (*)			

[*Si responde que "No pagaría seguro más de esta cantidad" y el valor es de 0 €, pasar a U.5. Si responde que "Pagaría seguro esta cantidad" una cantidad de 15 € diarios (450 € mensuales), formular la pregunta U.6. En cualquier otro caso, saltar a las siguientes preguntas*].

María del Mar García Calvente, María del Río Lozano, Isabel Larrañaga Padilla. Cuestionario del Proyecto CUIDAR-SE: Estudio de seguimiento de la salud y la calidad de vida de mujeres y hombres cuidadores en Andalucía y País Vasco. Granada: Escuela Andaluza de Salud Pública; 2019.
Financiado por el Ministerio de Economía y Competitividad, Instituto de Salud Carlos III y Fondos FEDER (Expte. Nº PI12/00498).

W.5. [Sólo a quienes digan que “*No pagaría seguro más de esta cantidad*” y la cifra sea de 0 euros]

Ud. acaba de decir que está seguro de que no pagaría ni 0 euros por recibir el servicio de una hora al día de un cuidador profesional. ¿Podría Ud. decirme por qué motivo ha indicado dicha cifra por esa hora adicional de cuidados?

1. Cuidar a esa persona durante una hora más al día no me supondría una diferencia importante y, por tanto, me sería indiferente el que viniera o no un cuidador profesional
2. No confío en cuidadores profesionales
3. Es una cuestión de principios (no aceptaría pagar ni un sólo euro por un servicio público)
4. Otros motivos (especificar): …………………………………..

W.6. [Sólo a quienes respondan que “Pagaría seguro esta cantidad” una cantidad de 15 € al día]

Por favor, ¿podría indicar la cantidad diaria (o mensual) que Ud. consideraría como la máxima que estaría dispuesto a pagar por esa hora extra de cuidado profesional durante una hora más cada día?

...............€/día ó ………..€/mes

[Las personas encuestadas pueden indicar el importe diario o mensual, según les resulte más sencillo]

---OOO…

Por último, antes de pasar al último bloque de preguntas sociodemográficas, me gustaría que me respondiera a esta pregunta.

CR. 1. ¿Cree que la situación o las condiciones en las que cuida se ha visto modificada por la crisis económica?

1. Ha empeorado mucho
2. Ha empeorado ligeramente
3. No se ha modificado
4. Ha mejorado
9. NS/NC

María del Mar García Calvente, María del Río Lozano, Isabel Larrañaga Padilla. Cuestionario del Proyecto CUIDAR-SE: Estudio de seguimiento de la salud y la calidad de vida de mujeres y hombres cuidadores en Andalucía y País Vasco. Granada: Escuela Andaluza de Salud Pública; 2019.
Financiado por el Ministerio de Economía y Competitividad, Instituto de Salud Carlos III y Fondos FEDER (Expte. Nº PI12/00498).
MINISTERIO DE ECONOMÍA Y COMPETITIVIDAD
Instituto de Salud Carlos III
Unión Europea
Fondo Europeo de Desarrollo Regional
“Una manera de hacer Europa”
Andalucía se mueve con Europa

BLOQUE VII. SOCIODEMOGRÁFICAS

SD 1. Edad:

SD 2. ¿Cuál es su estado civil legal?
1. Soltero/a
2. Casado/a
3. Viudo/a
4. Separado/a
5. Divorciado/a

SD 3. ¿Convive actualmente en pareja?
1. Si
2. No

SD 4. ¿Cuál es su nacionalidad?
1. Española (Pasar a SD6)
2. Extranjera
3. Española y otra

SD 5. ¿De qué país es su nacionalidad?
1. Un país de la Unión Europea excepto Rumanía y Bulgaria (UE-25)
2. Rumanía o Bulgaria
3. Otro país de Europa
4. Canadá o EEUU
5. Ecuador
6. Colombia
7. Otro país de América
8. Un país de Asia
9. Marruecos
10. Otro país de África
11. Un país de Oceanía

SD 6. ¿Cuál es su situación laboral actual?
1. Trabaja
2. En paro y ha trabajado antes -------------- S.7. ¿Cuántos meses en el paro? _ _
3. Busca primer empleo
4. Jubilado/a
5. Dedicado/a principalmente a las labores del hogar (amo/a de casa)
6. Estudiante
7. Incapacidad / Invalidez permanente
8. Servicio militar /objeción
10. Otros (Especificar)
9. NS/NC

Si trabaja o ha trabajado anteriormente: pasar a SD 9

María del Mar García Calvente, María del Río Lozano, Isabel Larrañaga Padilla. Cuestionario del Proyecto CUIDAR-SE: Estudio de seguimiento de la salud y la calidad de vida de mujeres y hombres cuidadores en Andalucía y País Vasco. Granada: Escuela Andaluza de Salud Pública; 2019.
Financiado por el Ministerio de Economía y Competitividad, Instituto de Salud Carlos III y Fondos FEDER (Expte. Nº PI12/00498).

Si no trabaja ni ha trabajado anteriormente: pasar a SD 12

SD 9 ¿Cuál es o era la actividad principal de la empresa donde trabaja o trabajaba? _ _

SD 10. ¿Cuál es la ocupación que desempeña en la actualidad o la última que ha desempeñado? _ _ _

SD 11. ¿Cuál es la categoría profesional que tiene o tenía en la empresa donde trabaja o trabajaba?
1. Trabajador por cuenta propia, sin asalariados
2. Trabajador por cuenta propia, con 10 ó más asalariados
3. Trabajador por cuenta propia, con menos de 10
4. Gerente de una empresa con 10 ó más asalariados
5. Gerente de una empresa con menos de 10 asalariados
6. Capataz, supervisor o encargado
7. Otro asalariado
9. NS/NC

SD 12. ¿Cuál es el nivel más alto de estudios finalizados?
1. No sabe leer ni escribir
2. No ha estudiado pero sabe leer y escribir
3. Estudios primarios (hasta 5º EGB, ingreso)
4. EGB completa (8º) o similar (bachillerato elemental)
5. Estudios de FP I
6. Estudios de FP II
7. Estudios secundarios (BUP, B. Superior)
8. Estudios universitarios de grado medio
9. Estudios universitarios de grado superior
10. Otros estudios
99. NS/NC

SD 13. Por favor, indique el intervalo en el que están comprendidos los ingresos totales netos de su hogar, sumando todas las fuentes (si existe más de una) y deduciendo las retenciones a cuenta por impuestos, cotizaciones sociales y otros pagos asimilados (MOSTRAR TARJETA)
1. Hasta 499 €
2. De 500 a 999 €
3. De 1.000 a 1.499 €
4. De 1.500 a 1.999 €
5. De 2.000 a 2.499 €
6. De 2.500 a 2.999 €
7. De 3.000 a 4.999 €
8. Más de 5.000 €
9. NS/NC

Financiado por el Ministerio de Economía y Competitividad, Instituto de Salud Carlos III y Fondos FEDER (Expte. Nº PI12/00498).
MINISTERIO DE ECONOMÍA Y COMPETITIVIDAD
Instituto de Salud Carlos III
Unión Europea
Fondo Europeo de Desarrollo Regional
"Una manera de hacer Europa"
Andalucía se mueve con Europa
